# PLIN2 promotes colorectal cancer progression through CD36-mediated epithelial-mesenchymal transition

**DOI:** 10.1038/s41419-025-07836-1

**Published:** 2025-07-10

**Authors:** Fan Yang, Ying Li, Xue Shang, Yun Zhu, Wenting Hou, Yi Liu, Qing Hua, Zhirong Sun

**Affiliations:** 1 Department of Anesthesiology, Fudan University Shanghai Cancer Center; Department of Oncology, Shanghai Medical College Fudan University, Shanghai, 200032 China; 2 Department of Anesthesiology, Zhongshan Hospital Fudan University, Shanghai, 200032 China

**Keywords:** Lipid signalling, Tumour biomarkers, Cancer microenvironment

## Abstract

Colorectal cancer (CRC) is one of the most common malignant tumors with high incidence and mortality. The challenge remains to construct reliable prognostic prediction models and to further elucidate the key molecular mechanisms of tumor progression. To address this, we performed WGCNA based on 120 immune cell expression profiles from GEO sources to obtain a collection of monocytes/macrophages-related genes. The prognostic model was constructed by univariate survival analysis and LASSO regression analysis. Then, the prognostic model was validated by Multivariate Cox regression, Kaplan–Meier survival analysis and ROC analysis. In this prognostic model, we identified that PLIN2 has a potential value for CRC prognosis. PLIN2 expression in monocytes/macrophages was verified by scRNA-seq datasets and spatial transcriptome datasets, and PLIN2 was found to promote macrophage transformation to M2 subtype. Clinical specimens and tissue microarrays confirmed the differential expression and prognostic value of PLIN2 in CRC patients. Functional experiments demonstrated that PLIN2 gene overexpression promoted the proliferation, migration and invasion of CRC cells and significantly facilitated tumor growth in vivo. Mechanistically, we revealed that CD36 is a potential downstream target gene of PLIN2. The CD36 inhibitor Sulfo-N-succinimidyl Oleate significantly reversed PLIN2-induced proliferation, migration, invasion, and EMT activity of CRC cells in vitro and in vivo. Immunoprecipitation and immunofluorescence experiments confirmed that PLIN2 could interact with CD36. PLIN2 stabilized CD36 protein expression by inhibiting the proteasomal degradation pathway, thereby promoting CD36-mediated EMT activity. Overall, our study highlights that the PLIN2/CD36 axis regulates EMT activity and CRC progression, suggesting that interventions in this signaling pathway may offer a promising therapeutic approach to CRC progression.

**Figure:**
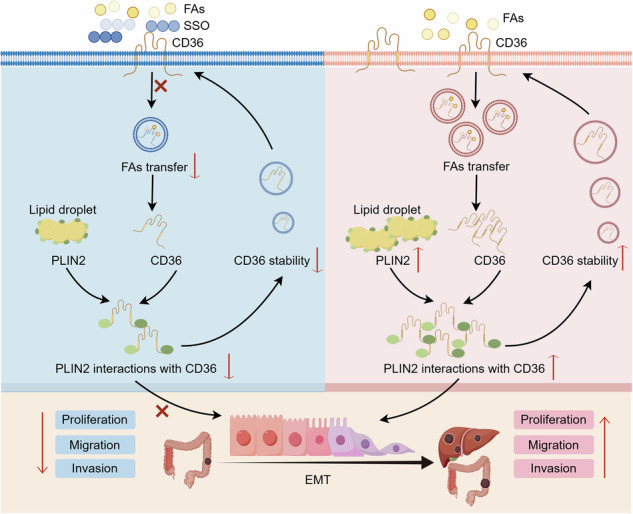
Schematic diagram elucidating the role of PLIN2 in CRC by Figdraw. FA is transported into the cell via CD36-mediated endocytosis. In CRC cells, PLIN2 promotes stability of CD36 and interacts with CD36 to activate the EMT process. However, the CD36 inhibitor SSO inhibits the binding of FAs to CD36 and attenuates its endocytosis, thereby reversing the PLIN2-mediated EMT process. Ultimately, the PLIN2-induced enhancement of CRC cell proliferation, migration, and invasion is attenuated by the CD36 inhibitor SSO.

Schematic diagram elucidating the role of PLIN2 in CRC by Figdraw. FA is transported into the cell via CD36-mediated endocytosis. In CRC cells, PLIN2 promotes stability of CD36 and interacts with CD36 to activate the EMT process. However, the CD36 inhibitor SSO inhibits the binding of FAs to CD36 and attenuates its endocytosis, thereby reversing the PLIN2-mediated EMT process. Ultimately, the PLIN2-induced enhancement of CRC cell proliferation, migration, and invasion is attenuated by the CD36 inhibitor SSO.

## Introduction

Colorectal cancer (CRC) is one of the major malignant neoplasms in humans, with the third and second highest incidence and mortality rates, respectively. According to statistics, 1,926,118 new cases of CRC were diagnosed in 2022, and more than 900,000 patients died of CRC [1]. Surgical resection is the principal method of treatment for CRC. In recent years, with the emergence of new methods such as radiotherapy, chemotherapy and immunotherapy, significant progress has been made in the management of CRC. However, the prognosis of CRC patients is still unsatisfactory due to local invasion and distant metastasis [2–4]. However, more than half of patients with advanced disease will develop metastases two years after the primary tumor is removed [5].

CRC progression involves multiple mechanisms, including mutations in key oncogenes, activation of aberrant signaling pathways and alterations in the tumor microenvironment [6]. Among these, epithelial-mesenchymal transition (EMT) plays an important role in CRC progression, metastasis and drug resistance [7–10]. EMT is a condition in which epithelial cells lose their polarity and intercellular junctions but gain the properties of mesenchymal cells, with an increased ability to migrate and invade [11]. EMT is characterized by the down-regulation of epithelial markers (e.g., E-cadherin and occludin) and the up-regulation of mesenchymal markers (e.g., N- cadherin and vimentin) [12, 13]. These changes allow cancer cells to cross the basement membrane, enter the bloodstream or lymphatic system, and form metastatic foci at distant sites, such as the liver [11].

Surgical resection is the primary treatment for CRC. Perioperative fasting and abstinence from food and drink are routine surgical practices aimed at reducing the risk of gastric contents reflux aspiration and postoperative complications [14, 15]. Our previous study found that fasting inhibited M2 polarization in macrophages, which in turn inhibited tumor growth by reducing adenosine levels in the CRC tumor microenvironment [16]. In addition to this, we also found that fasting upregulated the cholesterol-producing gene FDFT1, which led to a decrease in glycolysis in CRC [17]. Prolonged fasting and abstinence from food and drink may lead to significant changes in the metabolic state of the patient, especially lipid metabolism [18]. Lipids perform important biological functions in the body, including storing energy, forming cell membranes, and acting as signaling molecules. However, recent studies have found that lipids are strongly associated with the development of malignant tumors, including CRC [19–21]. In addition to directly affecting the biological function of malignant tumors, it has also been found that lipids can remodel the tumor immune microenvironment, which in turn promotes malignant tumor development [22].

The family of peripheral lipoproteins (PLINs) is specifically associated with the surface of lipid droplets, contributes to the stabilization of the lipid droplets surface [23]. There are five different PLINs (PLIN1-5) that work together in different ways to regulate lipid metabolic processes [24, 25]. PLIN2 is a member of the family of PLINs, also known as adipose differentiation-associated proteins (ADRPs), which play a key role in lipid droplets formation and maintenance of lipid homeostasis [26]. In recent years, there has been increasing evidence linking PLIN2 to malignant tumors, including breast, liver, kidney, lung and oral cancer [27–34]. Matsubara et al. suggested that PLIN2 might serve as a screening marker for early CRC by using plasma samples from 43 patients [35]. However, the expression pattern of PLIN2 in CRC tumor parenchyma and tumor microenvironment and its molecular mechanism in CRC are not clear.

After infiltration into tumor tissue, circulating monocytes differentiate into tumor-associated macrophages (TAMs) driven by local signals (e.g. cytokines secreted by tumor cells). TAMs are central regulators in the tumor immune microenvironment, and are multifunctional and highly malleable, directly influencing tumor progression and immunotherapeutic response through the M1-M2 phenotypic transition [36–38]. Although PLIN2 has been shown to be expressed in monocytes/macrophages, its relationship with M2 polarization has not been clarified [23, 34, 39, 40].

CD36 is a transmembrane glycoprotein that is primarily responsible for fatty acids (FAs) recognition and cellular translocation across membranes, which help cells obtain energy and build cell membranes [41]. In tumor cells, high expression of CD36 promotes fatty acid uptake and metabolic reprogramming, which correlates with malignant tumor biological behaviors [42]. In addition, CD36 can promote EMT through multiple mechanisms [43–45].

In this study, we constructed a risk model for CRC and identified PLIN2 as a potential prognostic biomarker. Mechanistically, PLIN2 accelerates CRC progression via a dual mechanism: it induces macrophage reprogramming towards the tumor-promoting M2 phenotype, while simultaneously triggering the CD36-dependent EMT cascade in CRC cells.

## Methods

### Tissues collection

Tissue samples from 96 colorectal cancer (CRC) patients, including both cancerous and paired adjacent non-cancerous tissues, were collected between June 2012 and December 2013 from patients who underwent surgical treatment at Fudan University Shanghai Cancer Center. All diagnoses were confirmed independently by two pathologists. The study was approved by the Ethics Committee of Fudan University Shanghai Cancer Center (approval number: 2408-Exp059), and informed consent was obtained from all participants prior to sample collection. Oncologic staging was conducted according to the American Joint Committee on Cancer (AJCC) Staging Manual.

### Data sources and pre-processing

Eight datasets related to immune cell lines were downloaded from the GSE Expression Omnibus, including GSE71274, GSE63327, GSE28491, GSE28490, GSE222156, GSE152215, GSE116660, and GSE182528. Batch effects between different datasets were removed using the ‘removeBatch Effect’ function of the ‘limma’ package in R. The probe IDs were then converted to gene symbols according to the annotation file.

Bulk RNA-seq data and clinical information from The Cancer Genome Atlas (TCGA) datasets of colon adenocarcinoma (COAD) and rectum adenocarcinoma (READ) were integrated to construct the TCGA-CRC cohort (*n* = 596), which was utilized for subsequent analyses. Samples of normal tissue and samples with missing follow-up information were excluded. Probes were converted to gene symbols based on annotation files. For the expression of multiple gene symbols, the median value was considered.

### Identification of monocyte marker genes

The 120 expression profiles from the immune cell dataset were clustered using hierarchical clustering after removing the batch effects, with distances between genes calculated using Pearson’s correlation coefficient. Additionally, a weighted co-expression network was constructed using the Weighted Correlation Network Analysis (WGCNA) in R software (v4.2.2).

The TCGA-CRC dataset was utilized to build a univariate Cox proportional hazards regression model for each monocytes/macrophages related gene, incorporating survival data using the ‘coxph’ package in R. The threshold for identifying prognostic genes was set at *p*-value < 0.05, leading to the selection of monocytes/macrophages-related genes related to CRC prognosis. We then corrected the p-values with a false discovery rate (FDR) correction. Lasso regression analysis was then conducted to refine the risk model by reducing the number of genes.

To determine the optimal cutoff value for PLIN2 expression, we used the surv_cutpoint function provided by the survminer package in R (version 4.2.2). After determining the optimal survival-related cut-off value for PLIN2 expression, we further used the survdiff function in R to statistically test the survival difference between the high and low expression groups and calculate the p-value of the log-rank test. Subsequently, the survival curves were visualized using the ggsurvplot function to visualize the survival differences between the two groups.

### The Cancer Genome Atlas (TCGA) analysis and Gene Set Enrichment Analysis (GSEA)

Samples from the Genotype-Tissue Expression (GTEx) database were used to supplement the TCGA-CRC database, yielding a total of 942 CRC samples and 389 normal samples. The expression levels of PLIN2 in CRC samples were compared to those in normal samples. GSEA was conducted to elucidate the mechanism of action of PLIN2 in CRC. Correlation coefficients between each gene and PLIN2 in the TCGA-CRC dataset were used as weights for GSEA enrichment analysis. The annotated gene set (h.all.v2023.2.Hs.entrez.gmt) was selected as the reference gene set. The normalized enrichment score (NES) and p-value indicated the significance of the association between gene sets and pathways.

### Single-cell RNA sequencing (scRNA-seq) analysis

The scRNA-seq data of CRC tissues were extracted from five GEO datasets (EMTAB8107, GSE139555, GSE146771_10X, GSE146771_Smartseq2, GSE166555) and subjected to analyze in the Tumor Immune Single-cell Hub (TISCH) database (http://tisch.comp-genomics.org/home/). The single-cell level expression matrix is subjected to normalization using the “NormalizeData” method within the “Seurat” package, aiming to standardize the raw counts in each cell. For each dataset, a consistent analytical algorithm was employed, encompassing processes such as quality control, clustering, and cell-type annotation.

### Spatial transcriptomics (ST) analysis

We analyzed the spatial transcriptomics data from Cancer Discovery (2022) 12 (1): 134–153 (http://www.cancerdiversity.asia/scCRLM) using the SORC (Spatial Omics Resource for Cancer, http://bio-bigdata.hrbmu.edu.cn/SORC) online platform [46]. SORC is an integrated bioinformatics resource designed for spatial transcriptomics analyses, offering a variety of visualization, clustering and differential expression analysis tools. Datasets are processed and analyzed within the platform following recommended processes, and results are visualized and interpreted according to the platform’s built-in functionality [47].

### Molecular docking

The structures of PLIN2 (Uniprot Q99541) and CD36 (Uniprot P16671) were downloaded from the AlphaFold Protein Structure Database. Protein docking predictions were obtained using GRAMM. The results were further analyzed using PDBePISA to investigate the docking sites (hydrogen bonds and salt bridges) between the receptor and ligand proteins. Visualization of the results was performed using PyMOL software.

### Animal studies

All in vivo experiments were performed using 4 ~ 6 weeks old male BALB / c nude mice. The mice were purchased from Changzhou Cavins Laboratory Animal Co., Ltd. and were placed in a room with controlled room temperature, and fed and watered freely under diurnal alternation. All the animal protocols were approved by the Experimental Animal Ethics Committee of Shanghai SINOGENE Life Technology Co., Ltd (approval number: XNG201-2407-001) and conformed to the principal guidelines of the Guide for the Care and Use of Laboratory Animals (8th edition, National Academies Press). In the xenograft model, nude mice were randomly divided into Lv-Control, Lv-PLIN2, and Lv-PLIN2 + SSO groups (*n* = 6 in each group). 5 × 10^7^ SW480-Lv-Control or SW480-Lv-PLIN2 cells were suspended in 200 μl PBS and injected subcutaneously in mice. When tumors grew to day 16, the PLIN2 + SSO group was injected intraperitoneally with the CD36 inhibitor SSO at a dose of 50 mg/kg/day. The remaining groups were given an equal volume of 0.1% dimethyl sulfoxide (DMSO) solution. Tumor size was measured every 4 days and calculated as V = 0.5 × (length) × (width)^2^. All mice were executed on the 28th day after inoculation, and tumor tissues were collected, photographed and weighed.

In the orthotopic CRC model, nude mice were anesthetized with isoflurane gas. Nude mice were randomly divided into Lv-Control, Lv-PLIN2, and Lv-PLIN2 + SSO groups (*n* = 5 in each group). The skin was incised to expose the abdominal cavity, and the cecum was exposed. 4 × 10^6^ SW480-Lv-Control or SW480-Lv-PLIN2 cells were suspended in 50 μl of PBS and injected into the intermucosal muscularis layer, and the cecum was reset and the abdominal cavity was closed by applying pressure with a sterile cotton swab for 1–2 min after the injection was finished and the needle was withdrawn. When the tumor grew to day 10, the PLIN2 + SSO group was injected intraperitoneally with the CD36 inhibitor SSO at a dose of 50 mg/kg/day. The remaining groups were given an equal volume of 0.1% DMSO solution. All mice were executed on day 21 post-inoculation, and tumor tissues were collected, photographed and weighed.

### Cell culture

The human CRC cell lines RKO and SW480 were obtained from the Type Culture Collection of the Chinese Academy of Sciences. THP-1 cells were purchased from the American Type Cultrue Collection (ATCC). RKO and SW480 cells were cultured in Dulbecco’s Modified Eagle Medium (DMEM) (Gibco, USA) supplemented with 10% fetal bovine serum (Gibco, USA) and 1% penicillin/streptomycin (Gibco, USA). THP-1 cell lines were cultured in RPMI 1640 (Gibco, USA), supplemented with 10% fetal bovine serum (Gibco, USA) and 1% penicillin/streptomycin (Gibco, USA).

### Stable transfection

To establish cell lines stably overexpressing PLIN2, a pcDNA3.1-PLIN2 plasmid was designed and synthesized by Guangzhou GeneReal Co. (Guangzhou, China) for PLIN2 gene overexpression, along with a negative control. Polybrene (6 μg/mL) was used to enhance lentivirus transfection efficiency, and puromycin (10 μg/mL) was used to select stably expressing cell lines.

### Transient transfection

siRNAs targeting PLIN2 were designed and synthesized by GeneCreate Co. (Shanghai, China). Transfection of siRNA into RKO and SW480 cells was performed using the Lipo6000 transfection reagent (Beyotime Biotechnology, China) according to the manufacturer’s instructions.

### RNA isolation and quantitative real‑time PCR (qRT‒PCR)

RNAs from RKO and SW480 cells were isolated using TRNzol reagent (Tiangen Biotech, Beijing, China) with reference to the manufacturer’s protocol. The RNAs were reverse transcribed into cDNA by using PrimeScript FAST RT reagent Kit with gDNA Eraser (Takara, Beijing, China), followed by qRT‒PCR. It was conducted as stated in the manual of TB Green^®^ *Premix Ex Taq*^™^ II FAST qPCR (Takara, Beijing, China) with an Applied Biosystems 7300 Detection System (Applied Biosystems®, CA). The primers used are shown in Table S1.

### Western blot analysis

Total protein was extracted from treated RKO and SW480 cells, and protein concentrations were determined using a BCA protein assay kit (Epizyme, Shanghai, China). Equal amounts of protein samples were separated by 10% SDS-PAGE and transferred onto 0.45 μm PVDF membranes (Epizyme, Shanghai, China). Membranes were blocked with 5% non-fat milk for 2 h at room temperature and then incubated with primary antibodies overnight at 4 °C. After incubation with secondary antibodies, chemiluminescent signals were detected using a chemiluminescence imaging system and quantified with ImageJ software. The antibodies used are shown in Table S2.

### Flow cytometry (FC)

To detect macrophage polarization, PLIN2 was stably transfected in THP-1 cells, cells were treated with phorbose-12-myristose-13-acetate (PMA) for 12 h. Cells were collected and fixed with paraformaldehyde (PFA) overnight at 4 °C. After centrifugation, cells were resuspended in flow cytometry buffer and stained with anti-CD86, anti-CD206 antibody for 30 min at room temperature. The antibodies used are shown in Table S2.

### Co-immunoprecipitation (Co-IP) assay

RKO or SW480 cells were lysed with IP lysis buffer (Beyotime Biotechnology, China) on ice for 30 min. To study the interaction between endogenous PLIN2 and CD36, proteins were incubated overnight at 4 °C with primary antibodies and protein A/G- agarose beads (Beyotime Biotech, China). The beads were then collected using a magnetic rack, washed three times with wash buffer, and resuspended in loading buffer. Finally, the beads were heated at 95 °C for 5 min and the supernatant was taken for SDS - PAGE. For SDS-PAGE analysis, the input samples were loaded at 25% of the total volume, whereas the IgG and IP samples were loaded at 100%. Then transfer the proteins to PVDF membranes for further analysis. The antibodies used are shown in Table S2.

### Immunohistochemica (IHC) staining

For tumor tissues collected from the xenograft model and orthotopic CRC model, tumor tissues were embedded in paraffin and cut into 4 μm sections. After the sections were dewaxed and hydrated, the tissues were treated with 3% H_2_O_2_ to block the presence of endogenous peroxidase. Antigen repair was performed using citrate buffer (pH 6.0) or TE buffer (pH 9.0). To prevent nonspecific labelling, normal goat serum was used for containment. Primary antibodies were then applied to the tissues and incubated at 4 °C overnight. Diaminobenzidine was used for color development and hematoxylin was used as a counterstain. Images were acquired under a light microscope and positively stained tumor cells were analyzed.

For CRC tissue microarrays, after immunohistochemical staining, PLIN2 staining was scored according to the intensity of staining and the percentage of positive cells. Staining intensity was scored as follows: 0 = no color; 1 = pale yellow; 2 = light brown; 3 = dark brown. The percentage of immunopositive tumor cells (number of positively labelled tumor cells/total number of tumor cells) was scored as follows: 0, <5% positive cells; 1, 6–25% positive cells; 2, 26–50% positive cells; 3, 51–75% positive cells; 4, >75% positive cells. The composite score was calculated as the product of the intensity of staining and the mean percentage of positive cells. The antibodies used are shown in Table S2.

### Immunofluorescence (IF) staining

RKO and SW480 cells were seeded into 35 mm glass-bottom culture dishes and fixed with 4% paraformaldehyde. After washing three times with PBS, the cells were blocked with 10% BSA solution at room temperature for 2 h. Primary antibodies were then added simultaneously and incubated for 12 h at 4 °C. After incubation with a 594-conjugated anti-rabbit IgG antibody for 2 h, the cells were labeled with 4’,6-diamidino-2-phenylindole (DAPI). Confocal laser scanning microscopy was performed using a confocal microscope. The antibodies used are shown in Table S2.

### Cell counting kit-8 (CCK-8) assay

Pretreated cells were seeded into a 96-well plate. RKO and SW480 cells were incubated with CCK-8 reagent (Biosharp, China) at 37 °C for 1 h, and absorbance was measured at 450 nm using a microplate reader (BioTek, Vermont, USA) at the appropriate time points (1, 2, 3, 4, or 5 days).

### Wound-healing assay

RKO and SW480 cells were seeded into 6-well plates and cultured until they reached 100% confluence. The cells were then cultured in serum-free DMEM containing the CD36 antagonist SSO (100 μM) for 24 h. Monolayers of cells were scratched with a 200 μL sterile lance tip and washed three times with PBS. The plates were imaged at 0 and 24 or 48 h using an inverted fluorescence microscope.

### Transwell migration and invasion assay

RKO and SW480 cells were resuspended in serum-free medium containing the CD36 inhibitor SSO (100 μM). Cell suspensions were inoculated into the upper chamber of transwell inserts with an 8.0 μm pore size, either without Matrigel (for migration) or with Matrigel (for invasion) (Corning-Costar, Cambridge, MA, USA). The lower chamber was filled with 600 μL of complete medium containing 10% fetal bovine serum. After 24 h of incubation, the cells that had migrated or invaded were stained with crystal violet, photographed under a microscope, and counted.

### Statistical analysis

Data are presented as means ± SEM from at least three independent experiments. All statistical analyses were conducted using SPSS 26.0. Independent samples t-tests were used to compare individual datasets with control values, while one-way ANOVA was used to compare data across multiple groups. The Kaplan–Meier method was employed for overall survival analysis, with the log-rank test used to compare differences. Analysis of variance was applied to assess significant differences between groups under different conditions. *P* < 0.05 was considered statistically significant (*P < 0.05, **P < 0.01, ***P < 0.001, ****P < 0.0001, ^#^*P* < 0.05, ^##^*P* < 0.01, ^###^*P* < 0.001, ^####^*P* < 0.0001).

## Results

### Bioinformatics analysis identifies PLIN2 as a potential modulator of CRC

**Fig. 1:**
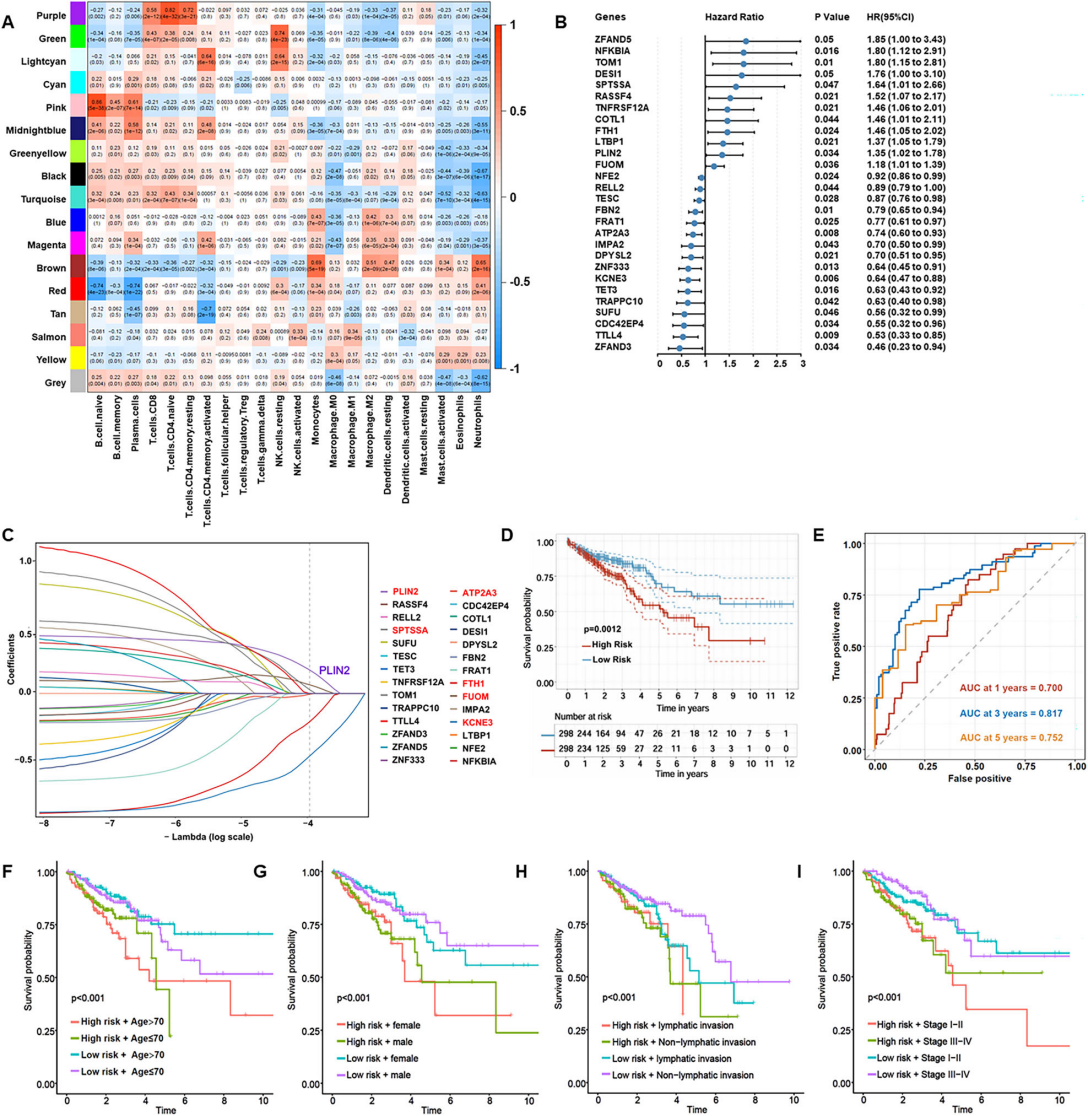
Construction and verification of a prognostic risk model based on monocytes/macrophages-related genes. **A** Correlation results between the 16 modules and each clinical phenotype. **B** Forest diagram of the intersected monocytes/macrophages-related genes significantly associated with the prognosis of CRC in TCGA-CRC cohorts. **C** Confidence intervals under each lambda. **D** Kaplan–Meier survival curve distribution of the 6-gene signature in TCGA-CRC database. **E** Time-dependent ROC analysis of 1-, 3-, and 5-year survival for CRC patients. **F–I** Survival analysis of our constructed model combined with clinical factors (age, gender, lymph node metastasis and stage).

In this study, we performed a multistep analysis to explore the prognostic model and potential modulators of CRC (Fig. S1). Firstly, eight immune cell datasets were obtained from the Gene Expression Omnibus (GEO) databases (Table S3), merged after batch effect removal (Fig. S2A), and clustered using hierarchical clustering on 120 expression profiles (Fig. S2B). To ensure a scale-free network, β = 8 was selected (Fig. S2C). Several studies found that perioperative immune dysfunction plays an important role in tumor relapse and our previous work focused on monocytes/macrophages dysregulation [16, 46, 48–53]. Among the 16 modules (Fig. S2D, Fig. 1A), the brown module, encompassing 1,204 genes, showed the most significant positive correlation with monocytes (Table. S4). We also found similar trends between these 1,204 genes and monocytes/macrophages in five scRNA-seq datasets (EMTAB8107, GSE139555, GSE146771_10X, GSE146771_Smartseq2, GSE166555) (Fig. S2E–I).

For further analysis, we performed univariate survival analysis in TCGA-CRC cohort. After excluding non-tumor, TNM staging and follow-up data unavailable samples, a total of 596 samples were included. The Benjamini-Hochberg method was applied to adjust for the FDR (Table. S5). Among the 1204 monocytes/macrophages-related genes, 28 genes were significantly associated with CRC prognosis (*P* < 0.05) (Fig. 1B). Next, Lasso-Cox regression analysis was performed to further refine the key genes. The trajectory of each independent variable was analyzed and found that as lambda increased, more independent variable coefficients approached zero (Fig. S3A). The optimal model was determined using ten-fold cross-validation and analysis of confidence intervals at various lambda values among the 28 genes. As shown in Fig. 1C, six genes, including three risk genes (PLIN2, FUOM, SPTSSA) and three protective genes (ATP2A3, KCNE3, FTH1) were achieved (lambda = 0.0190). Then, we calculated the risk scores of the 6 best candidate genes using the following equation: Risks Score=0.177 * PLIN2 + 0.065 * FUOM - 0.213 * FTH1 - 0.471 * KCNE3 + 0.028 * SPTSSA - 0.006 * ATP2A3 and their distribution was plotted (Fig. S3B).

Based on the prognostic risk scores calculated from the six-gene signature, patients were stratified into high- and low-risk groups. Kaplan–Meier analysis based on the six-gene risk score model revealed significantly worse survival in the high-risk group (*P* = 0.0012) (Fig. 1D). Time-dependent ROC results showed that the AUC of the model for predicting 1-, 3- and 5-year survival in CRC patients was 0.700, 0.817 and 0.752, respectively (Fig. 1E). Multivariate COX regression analysis showed that risk score was an independent prognostic factor for CRC patients (HR = 2.07, 95% CI, 1.38–3.09, *p* < 0.001; Fig. S3C). Based on the risk score and other clinical characteristics, we plotted the nomogram, which allows us to calculate the probability of survival rate at 1, 3, and 5 years of CRC patients (Fig. S3D). The risk model was combined with age, sex, lymph node metastasis, and AJCC TNM staging for survival analysis all supporting that the risk stratification model was stable (Fig. 1F–I). The prognostic robustness of the six-gene risk model was supported by time-dependent ROC and Kaplan–Meier survival analyses across the TCGA-CRC (10-fold cross-validation, *n* = 596, Fig. S3E–F), GSE17536 (*n* = 177, Fig. S3G–H), GSE17538 (*n* = 232, Fig. S3I, J), and GSE39582 (n = 577, Fig. S3K, L) cohorts. These results showed that the prognostic models exhibited robust prognostic stratification.

The identification of risk genes can help predict disease progression and prognosis since they are directly associated with poor prognosis [54]. Among the risk genes, PLIN2 had the highest risk coefficient, implying that PLIN2 may serve as a potential prognostic marker for CRC.

### PLIN2 is highly expressed in monocytes/macrophages and promotes M2 polarization

**Fig. 2:**
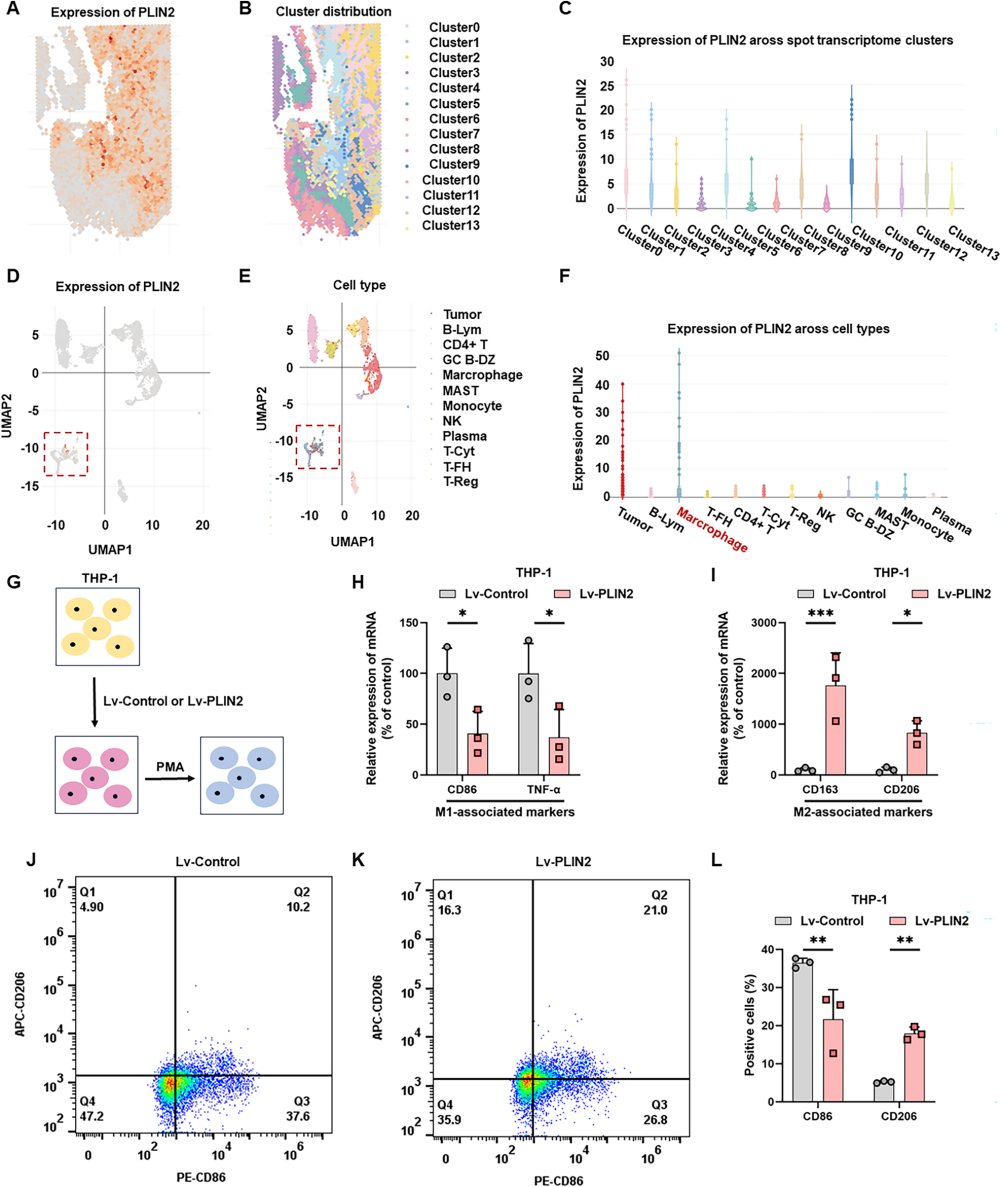
Distribution of PLIN2 in the spatial transcriptome and its regulation of M2 polarization in THP-1-derived macrophages. **A** Spatial distribution of PLIN2 expression across the tissue section. Color intensity indicates the relative expression level of PLIN2. **B** Unsupervised clustering of spatial transcriptomic spots. Each color denotes a distinct cluster identified based on global gene expression patterns. **C** Bar plot showing the expression of PLIN2 across the identified transcriptomic clusters. The y-axis indicates the normalized expression level, and the x-axis lists each cluster. **D** UMAP projection illustrating PLIN2 expression at the single-cell level. **E** UMAP projection annotated by cell type inferred from canonical marker genes. **F** Bar plot depicting PLIN2 expression levels across the major annotated cell types. The y-axis indicates normalized expression, and the x-axis lists each cell type. **G** Schematic diagram of the experimental procedure for induction of macrophage polarisation from THP-1 cells. **H–I** qRT-PCR analysis revealed that PLIN2 overexpression significantly downregulated M1 macrophage markers while upregulating M2-associated genes in THP-1 cells. **J–L** PLIN2 promotes the transformation of THP-1-derived macrophages to the M2 subtype as detected by flow cytometry. Data are shown as mean ± SD. *p < 0.05, **p < 0.01, ***p < 0.001.

This study above has identified PLIN2 as a monocytes/macrophages-associated gene by the bulk RNA-seq datasets, which was further confirmed in five scRNA-seq datasets (EMTAB8107, GSE139555, GSE146771_10X, GSE146771_Smartseq2, GSE166555) via UMAP visualization (Fig. S4A–E). Intersection analysis of five scRNA-seq cohorts identified 685 monocytes/macrophages-related genes (*P* < 0.05, Fig. S4F), including 19 with CRC prognostic significance (*P* < 0.05, Fig. S4G). Cross-validation of bulk and scRNA-seq datasets identified six overlapping genes, including PLIN2, confirming its association with monocytes/macrophages (Fig. S4H). In addition, based on spatial transcriptomic data from Wu et al. [55], Our results showed PLIN2 exhibits a distinct co-localization pattern between neoplastic cells and TAMs (Fig. 2A–F).

Previous studies have identified PLIN2 expression in monocytes/macrophages [23, 34, 39, 40]. However, the role of PLIN2 in the macrophage polarization remains largely unknown. To explore whether PLIN2 could affect macrophage polarization, we established THP-1 cells with stable overexpression of PLIN2, and after PMA-induced differentiation, M1 and M2-subtype markers were detected (Fig. 2G). qRT – PCR showed that THP-1 overexpression of PLIN2 not only up-regulated CD163 and CD206 (Fig. 2H, I), but also down-regulated CD86 and TNF - α. Flow cytometry also verified that PLIN2 promotes the transition of TAMs to M2 phenotype (Fig. 2J–L).

### PLIN2 is upregulated in CRC and associated with poor prognosis

**Fig. 3:**
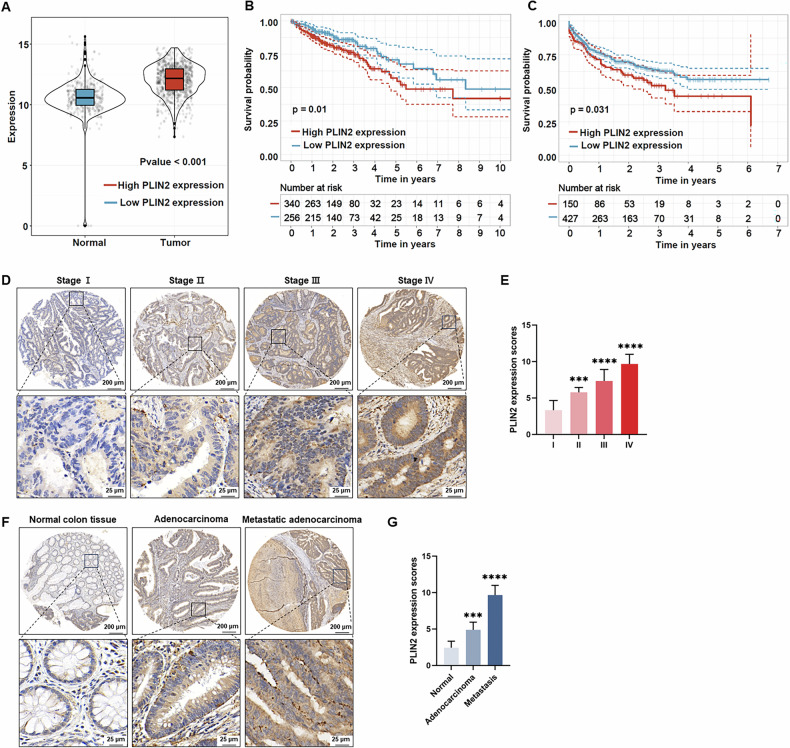
PLIN2 gene is highly expressed in CRC and correlates with poor prognosis. **A** The expression PLIN2 in CRC tissues (*n* = 942) and normal tissues (*n* = 389) analyzed in the TCGA-CRC and GTEx databases. **B, C** The Kaplan - Meier method for comparing the survival of patients with high and low PLIN2 expression in the TCGA-CRC dataset (*n* = 596) and GSE39582 dataset (*n* = 577). **D**, **E** PLIN2 expression in CRC tissues at different stages, from I to IV based on AJCC Cancer Staging Manual, were measured using IHC staining (**D**). PLIN2 expression scores were shown in (**E**). **F**, **G** PLIN2 expression in normal colon tissue, adenoma and metastatic adenocarcinoma was measured using IHC staining (**F**). PLIN2 expression scores were shown in (**G**). Data are shown as mean ± SD. ***p < 0.001, ****p < 0.0001.

The TCGA-CRC cohort was supplemented with normal samples from the GTEx database. Analysis revealed that PLIN2 expression levels were elevated in CRC samples (Fig. 3A). Kaplan-Meier curve analysis of the TCGA-CRC cohort, stratified by the optimal survival-related cut-off value for PLIN2 expression, demonstrated that patients with high PLIN2 expression had significantly shorter overall survival compared to the low-expression group (*P* = 0.01) (Fig. 3B). This prognostic significance of PLIN2 was further validated in the independent GSE39582 cohort, where classification based on the optimal survival-related cutoff again revealed a strong association between elevated PLIN2 expression and poorer clinical outcomes (*P* = 0.031) (Fig. 3C). Multi-cohort validation demonstrated robust prognostic capacity of PLIN2 expression, achieving time-dependent AUCs of 0.695–0.814 (1-year), 0.621–0.750 (3-year), and 0.626–0.754 (5-year) across TCGA-CRC (*n* = 594, 10-fold cross-validation), GSE17536 (*n* = 177), GSE17538 (*n* = 232), and GSE39582 (*n* = 577) cohorts (Fig. S4I–L). IHC staining of samples from 96 CRC patients, staged according to the AJCC Cancer Staging Manual, 7th edition, showed that PLIN2 expression was higher in advanced CRC stages (Fig. 3D, E). Moreover, PLIN2 expression gradually increased across paracancerous tissues, non-metastatic adenocarcinomas, and metastatic adenocarcinomas (Fig. 3F, G).

### PLIN2 facilitates malignant behaviors of CRC cells

We presently found that PLIN2 is highly expressed in both macrophages and tumor cells by spatial transcriptome, and found that PLIN2 promotes macrophage M2 polarization. However, extensive evidences have established that macrophage M2 polarization drives CRC progression [37, 52, 56]. Given our finding of high PLIN2 expression in CRC clinical samples, we subsequently focused our study on elucidating the role of PLIN2 on CRC tumor cells and related mechanisms.

**Fig. 4:**
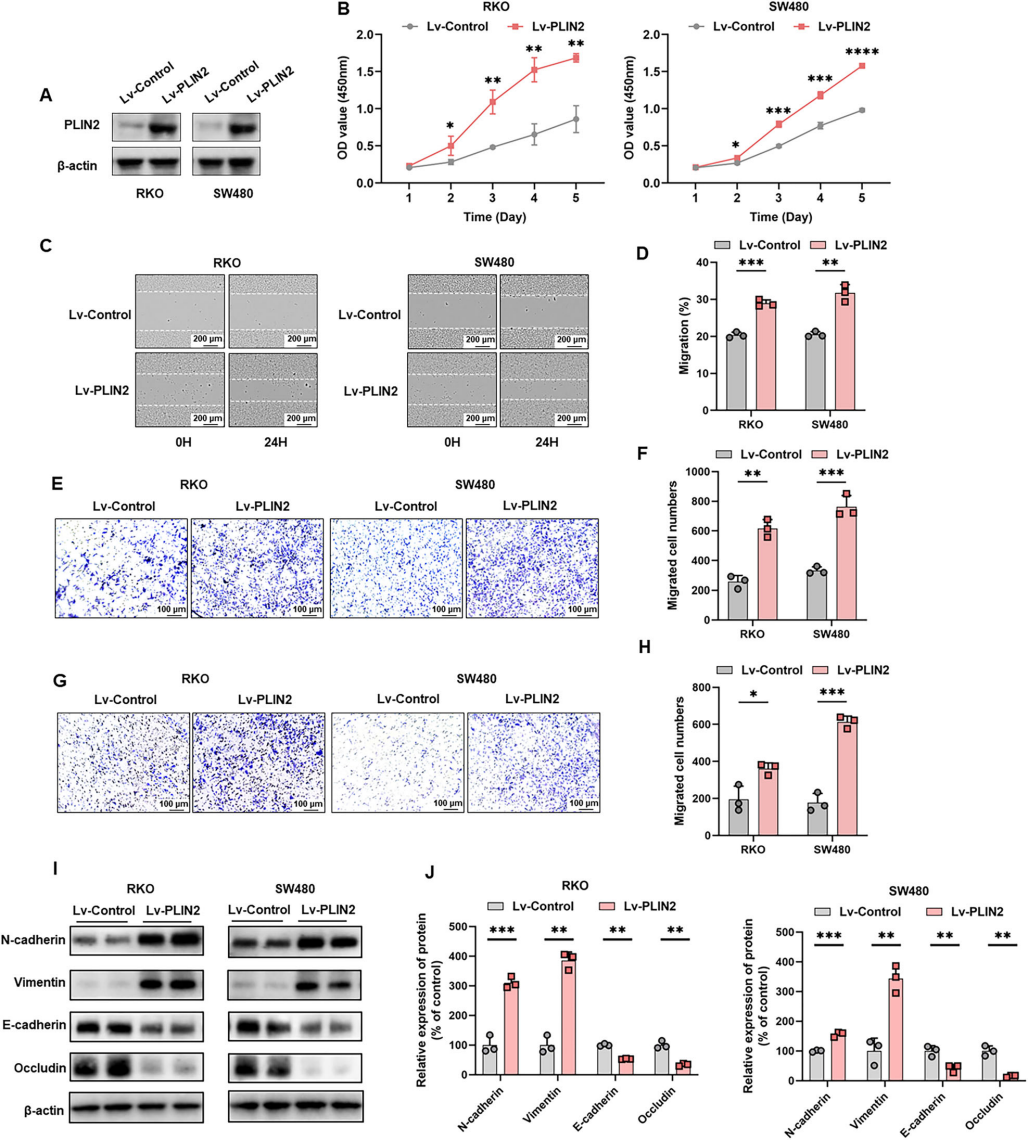
PLIN2 overexpression promotes the proliferation, migration and invasion and EMT of CRC cells in vitro. **A** Western blot analysis of the expression of PLIN2 after treatment with Lv-Control and Lv-PLIN2 in RKO and SW480 cells. **B** CCK8 assay was used to assess cell proliferation. **C**, **D** Wound-healing assay was used to assay cell migration. **E**, **F** Cell migration was determined by using transwell migration assays. **G**, **H** Cell invasion was determined by using transwell invasion assays. Cells invading through uncoated inserts and Matrigel-coated inserts were stained. **I**, **J** Western blot analysis showed that PLIN2 decreased the expression levels of the epithelial cell markers E-cadherin and occludin and increased the mesenchymal markers N-cadherin and vimentin in RKO and SW480 cells. Data are shown as mean ± SD. *p < 0.05, **p < 0.01, ***p < 0.001, ****p < 0.0001.

To investigate the biological function of PLIN2 in CRC cells, we established stable overexpression in RKO and SW480 cell lines. Western blot analysis confirmed successful overexpression in both cell lines (Fig. 4A). CCK-8 assays demonstrated that PLIN2 overexpression significantly promoted CRC cell proliferation compared to controls (Fig. 4B). The wound-healing assay showed that cells overexpressing PLIN2 exhibited significantly faster wound closure (Fig. 4C, D). Additionally, transwell assays revealed that PLIN2 overexpression markedly enhanced both migration (Fig. 4E, F) and invasion (Fig. 4G, H) capabilities of CRC cells.

In contrast, in the PLIN2 knockdown model, western blot analysis confirmed effective knockdown in RKO and SW480 cells (Fig. S5A). CCK-8 assays demonstrated that PLIN2 knockdown significantly inhibited CRC cell proliferation (Fig. S5B). The wound-healing assay showed that CRC cells with PLIN2 knockdown exhibited slower wound closure compared to controls (Fig. S5C, D). Furthermore, transwell assays indicated that PLIN2 knockdown led to a significant reduction in both migration (Fig. S5E, F) and invasion (Fig. S5G–H) abilities of RKO and SW480 cell lines.

### PLIN2 promotes EMT in CRC

To delve into the mechanism by which PLIN2 promotes CRC progression, GSEA was conducted using the TCGA-CRC database (Fig. S6A, B). Focusing on EMT, we examined EMT-associated markers, including E-cadherin, N-cadherin, occludin, and vimentin. Overexpression of PLIN2 in RKO and SW480 cells led to a marked reduction in the epithelial markers E-cadherin and occludin, while the mesenchymal markers N-cadherin and vimentin were significantly increased (Fig. 4I, J).

### PLIN2 stabilized CD36 protein by inhibiting the proteasome degradation pathway

**Fig. 5:**
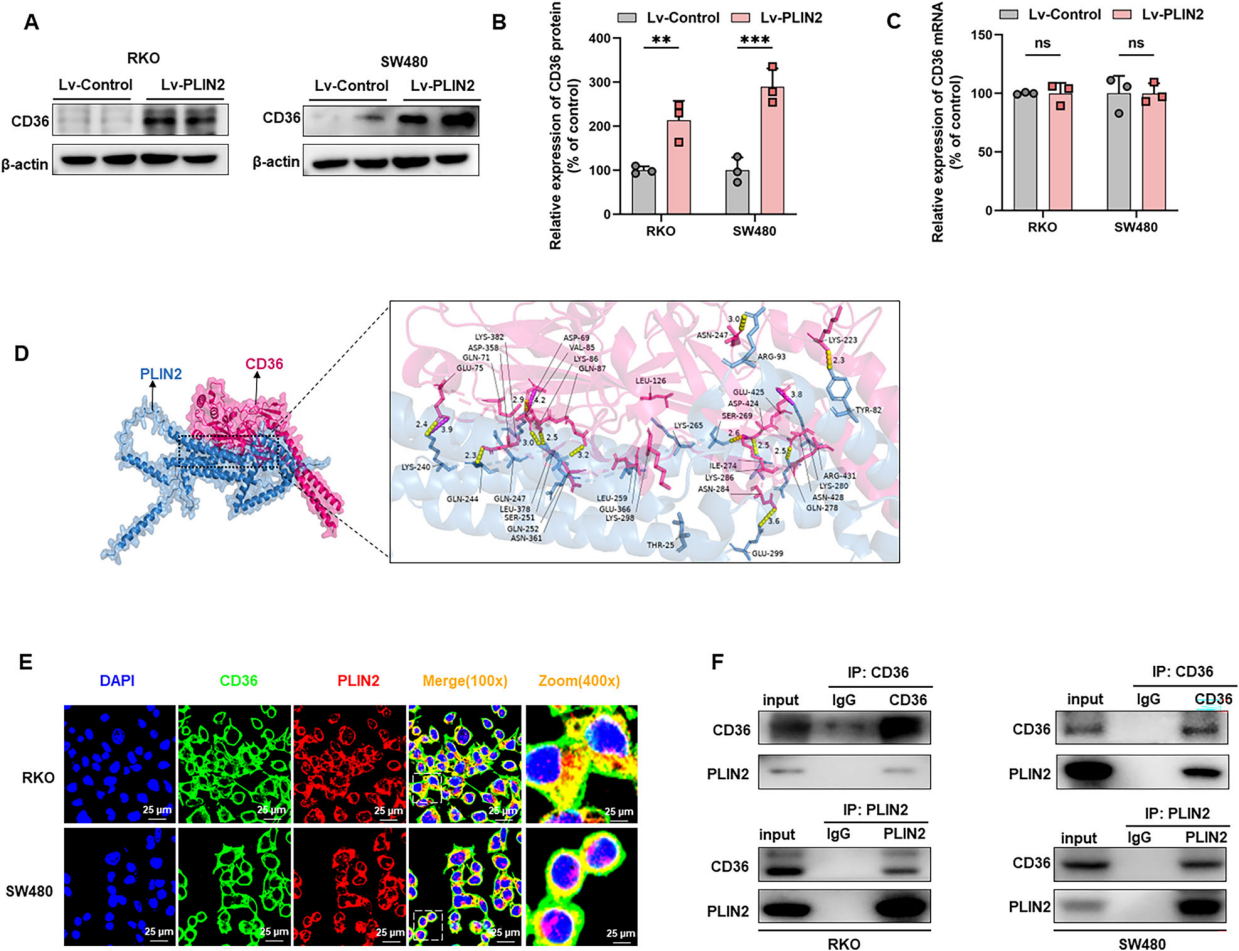
The interaction between PLIN2 and CD36. **A** Western blot analysis showed that PLIN2 increased the expression level of CD36 protein in RKO and SW480 cells. **B** Quantification of the protein expression of CD36 represented in (C). **C** qRT‒PCR showed that PLIN2 increased the expression level of CD36 protein in RKO and SW480 cells. **D** Molecular docking simulation of PLIN2 (blue) and CD36 (pink) (left). Amino acid sites and reciprocal hydrogen bonds (yellow) and salt bridges (purple) of PLIN2 and CD36 interaction (right). **E** Immunofluorescence assays were performed to detect co-localization of PLIN2 with CD36. **F** Co-IP showed a direct interaction between CD36 and PLIN2 in RKO and SW480 cells. Data are shown as mean ± SD. **p < 0.01, ***p < 0.001.

PLIN2, a lipid droplet-associated protein, has been implicated in tumor progression via lipid metabolic pathways [34]. Our prior in vitro experiments demonstrated that PLIN2 promotes CRC proliferation and metastasis through EMT. CD36, a lipid-associated transmembrane protein involved in FAs recognition and translocation, has been linked to tumor progression via EMT in various cancers, though its role in CRC remains unclear [41, 42, 57–59]. We analyzed the relationship between CD36 expression levels and prognosis in 596 CRC patients in the TCGA-CRC dataset. Our results found that high expression of CD36 was associated with poorer prognosis in CRC patients (*P* < 0.001) (Fig. S7A). In RKO and SW480 cell lines overexpressing PLIN2, CD36 protein levels were found to be significantly upregulated by western blot assay (Fig. 5A, B). On the other hand, overexpression of PLIN2 had no significant effect on CD36 mRNA levels according to qRT-PCR analysis (Fig. 5C).

Protein-protein interaction analysis using computer simulations suggested that PLIN2 and CD36 interact through salt bridges and hydrogen bonds (Fig. 5D). Immunofluorescence experiments confirmed that PLIN2 and CD36 co-localized in the cytoplasm of RKO and SW480 cells (Fig. 5E), a finding further supported by Co-IP experiments. In RKO and SW480 cell lines, PLIN2 was efficiently immunoprecipitated with CD36, and vice versa (Fig. 5F).

We found that PLIN2 affected CD36 protein expression levels without affecting CD36 mRNA expression levels in RKO and SW480 cells. These results suggest that PLIN2 promotes CD36 expression through post-transcriptional regulation. Next, we treated PLIN2 overexpressing or control RKO and SW480 cells with cycloheximide (CHX) (50 μg/ml) to block protein de novo synthesis. Our results showed that the rate of CD36 protein degradation was significantly lower in PLIN2 overexpressing cells than in control cells (Fig. S7B–E). These results suggest that PLIN2 overexpression increases the stability of CD36 protein in CRC cells. The proteasome and lysosomal pathways are the major routes of protein degradation in eukaryotic cells [60]. Wang et al. demonstrated that PLIN2 restricts apoptosis by decreasing ubiquitination of Bcl - 2 through the protease pathway [61]. Xia et al. showed that UCHL1 stabilizes CD36 protein expression via the protease pathway [62]. However, it is unknown whether PLIN2 can stabilize CD36 protein expression levels via the proteasome pathway. To investigate the involvement of the proteasome pathway in the protein degradation of CD36, we applied the proteasome inhibitor MG132 to PLIN2 overexpressing CRC cells. Our results showed that MG132 treatment reversed the degradation of CD36 in the control group, resulting in CD36 levels close to those of the PLIN2 overexpression group (Fig. S7F–H). However, treatment with chloroquine (CQ), an inhibitor of autophagy and the lysosomal pathway, did not reverse the degradation of CD36 caused by PLIN2 deficiency (Fig. S7I–K). This suggests that in RKO and SW480 cells, PLIN2 promotes CD36 stabilization by inhibiting the proteasomal degradation pathway, but not the lysosomal pathway.

### CD36 inhibition suppresses PLIN2-induced proliferation, migration, and invasion in CRC Cells

**Fig. 6:**
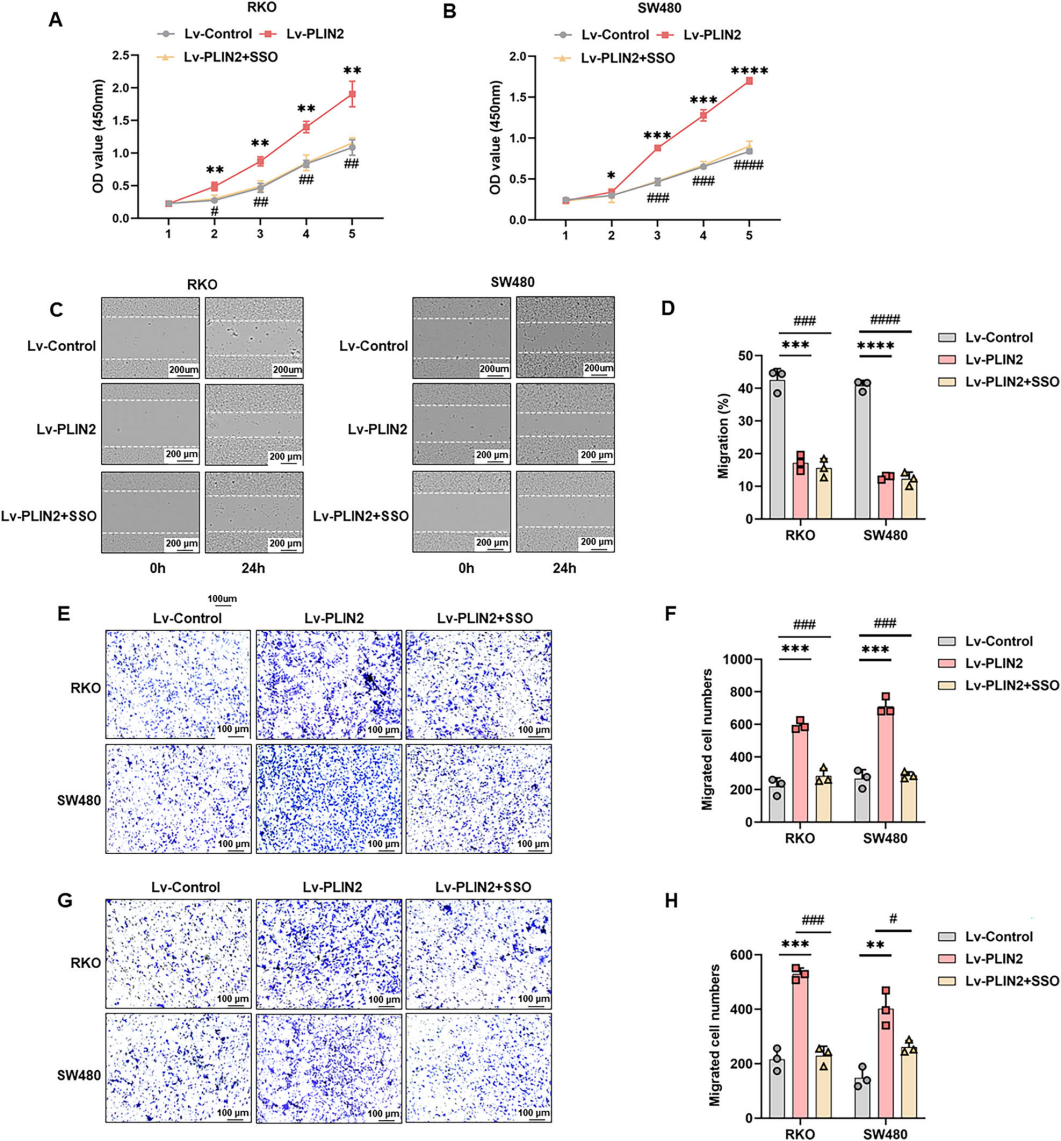
CD36 inhibition suppresses the proliferation in CRC cells induced by PLIN2 in vitro. **A, B** CCK8 assay was used to assess cell proliferation. **C, D** Wound-healing assay was used to assay cell migration. **E, F** Cell migration was determined by using transwell migration assays. **G, H** Cell invasion was determined by using transwell invasion assays. Cells invading through uncoated inserts and Matrigel-coated inserts were stained. Data are shown as mean ± SD. *p < 0.05, **p < 0.01, ***p < 0.001, ****p < 0.0001 (Lv-control vs Lv-PLIN2); ^#^*p* < 0.05, ^##^*p* < 0.01, ^###^*p* < 0.001, ^####^*p* < 0.0001 (Lv-PLIN2 vs Lv-PLIN2 + SSO).

To assess whether CD36 mediates PLIN2-induced CRC proliferation and metastasis, we treated RKO and SW480 cells overexpressing PLIN2 with 100 μM SSO for 24 h. The CCK-8 assay demonstrated that reversed the proliferative effects of PLIN2 overexpression (Fig. 6A, B). Similarly, SSO inhibited PLIN2-induced migration in wound-healing (Fig. 6C, D) and transwell migration assays (Fig. 6E, F). The invasive potential of CRC cells was also reduced by SSO treatment (Fig. 6G, H), indicating that CD36 inhibition counters the enhanced proliferation and metastatic capacity induced by PLIN2 overexpression.

### CD36 inhibition suppresses PLIN2-induced EMT in CRC cells

**Fig. 7:**
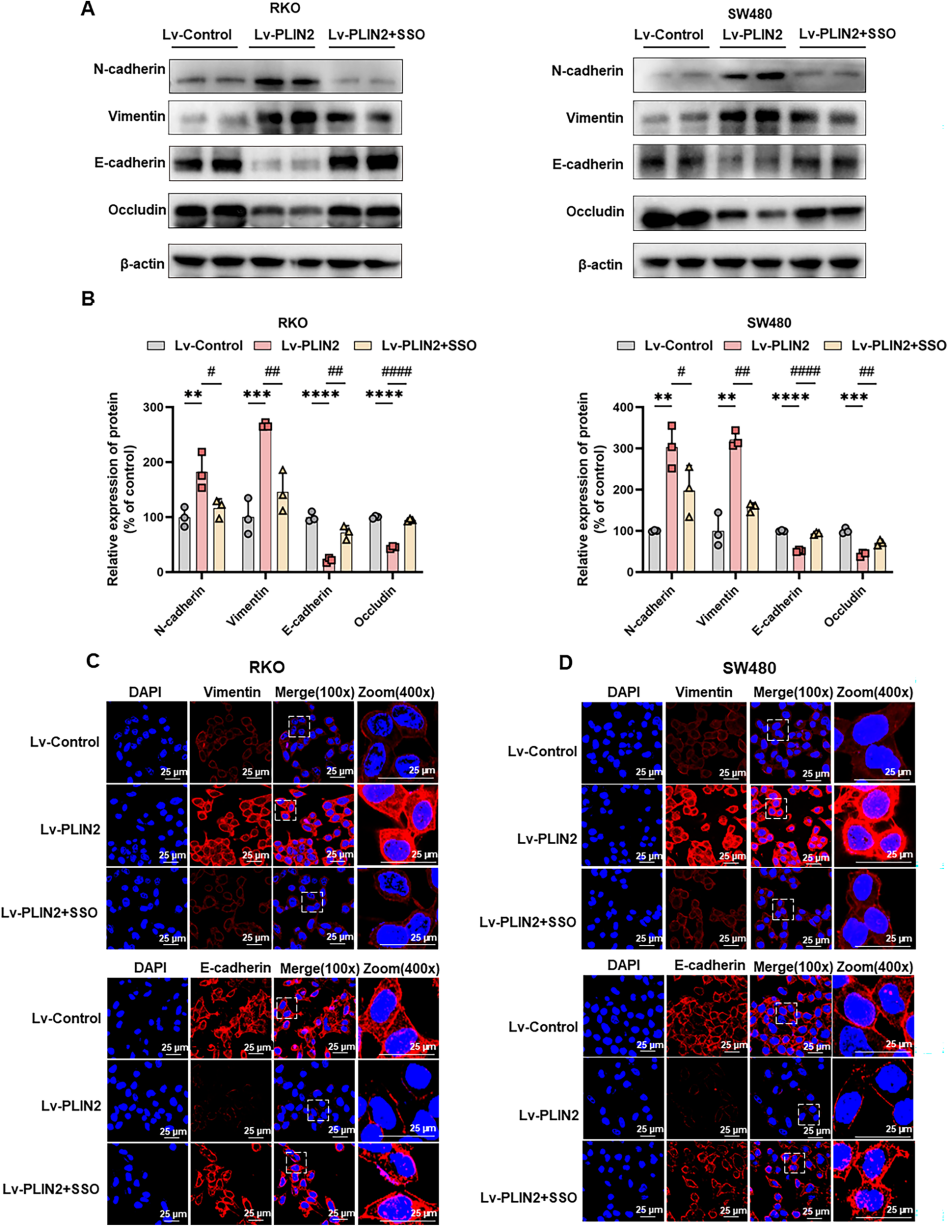
CD36 inhibition suppresses EMT in CRC cells. **A** Western blot analysis showed that CD36 inhibition decreased the expression levels of the epithelial cell markers E-cadherin and occludin and increased the mesenchymal markers N-cadherin and vimentin in RKO and SW480 cells compared with the Lv-PLIN2 group. **B** Quantification of the protein expression of E-cadherin, N-cadherin, Vimentin and Occludin represented in (**A**). **C**, **D** Immunofluorescence of E-cadherin and Vimentin in RKO and SW480 cells. Data are shown as mean ± SD. **p < 0.01, ***p < 0.001, ****p < 0.0001 (Lv-control vs Lv-PLIN2); ^#^*p* < 0.05, ^##^*p* < 0.01, ^####^*p* < 0.0001 (Lv-PLIN2 vs Lv-PLIN2 + SSO).

Finally, we examined whether PLIN2-induced EMT in CRC is mediated by CD36. Pretreatment of PLIN2-overexpressing CRC cell lines with 100 μM SSO for 24 h led to significant changes in EMT markers. Specifically, SSO incubation restored the levels of E-cadherin and occludin, which were reduced by PLIN2 overexpression (Fig. 7A, B). Meanwhile, the upregulation of N-cadherin and vimentin induced by PLIN2 was also reversed by SSO (Fig. 7A, B). These findings were confirmed by immunofluorescence assay to observe the expression of E-cadherin and vimentin after treatment with SSO (Fig. 7C, D).

These results suggest that CD36 inhibition effectively reverses the EMT process driven by PLIN2 overexpression in CRC cells.

### Inhibition of CD36 suppresses PLIN2-induced CRC progression in vivo

**Fig. 8:**
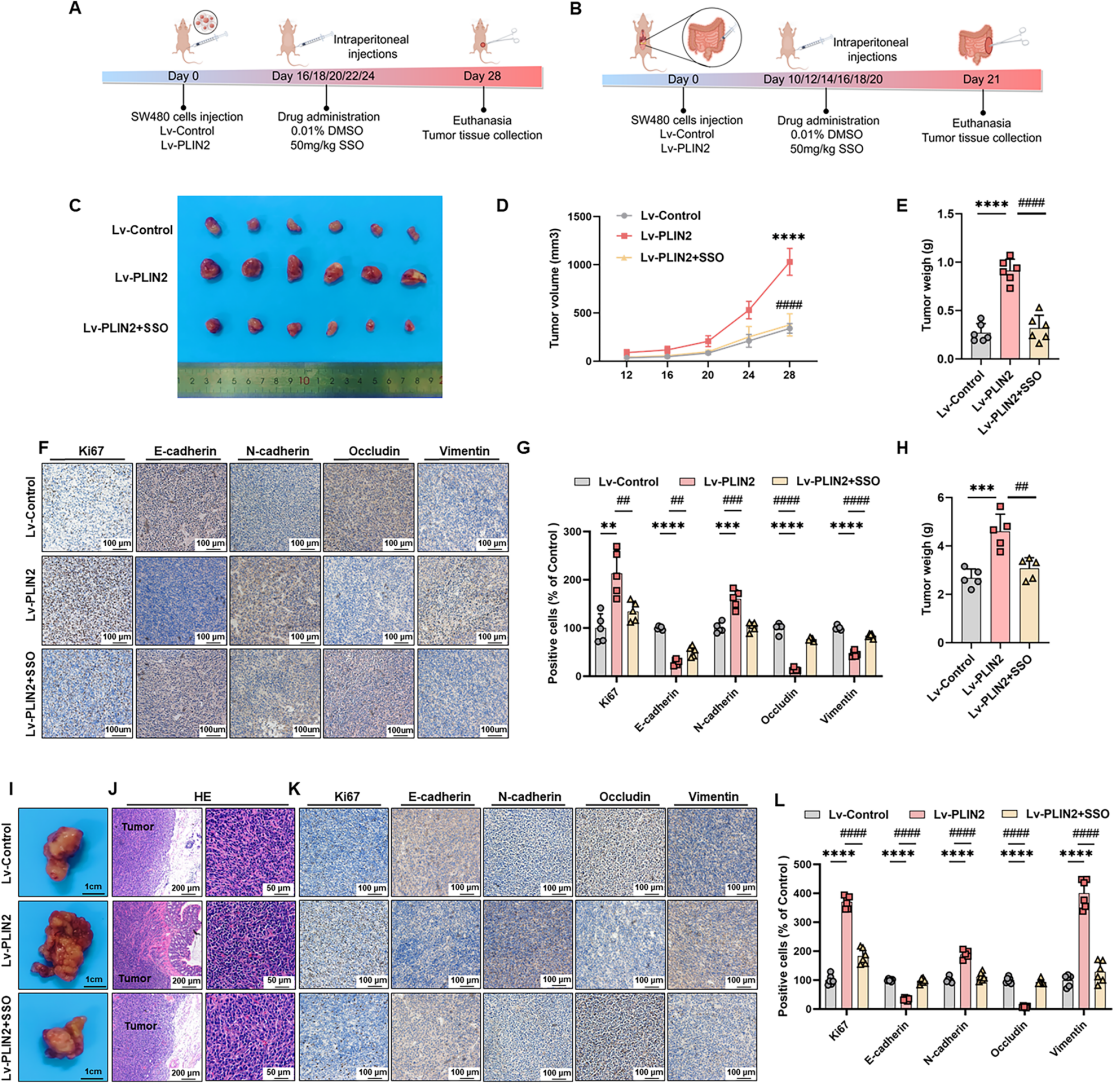
PLIN2 promotes CRC progression via CD36 in vivo. **A**, **B** Schematic diagram of the subcutaneous xenograft model (**A**) and CRC orthotopic model (**B**) by Figdraw. **C–E** Tumor tissue images (**C**), tumor volume statistical curves (**D**) and tumor weights (**E**) of Lv control, Lv-PLIN2, and Lv-PLIN2 + SSO groups of tumor-bearing mice. **F** Representative IHC staining images of E-cadherin, N-cadherin, occludin, and vimentin and Ki-67 in subcutaneous xenograft model tumor tissue sections of each group. **G** The statistical analysis of IHC staining in (**F**). **H, I** Tumor tissue images (**I**) and tumor weights (**H**) of Lv-control, Lv-PLIN2, and Lv-PLIN2 + SSO groups of CRC orthotopic model mice. **J** Representative HE staining images of CRC orthotopic model tumor. **K** Representative IHC staining images of E-cadherin, N-cadherin, occludin, vimentin and Ki-67 in CRC orthotopic model tumor tissue sections of each group. **L** The statistical analysis of IHC staining in (**K**). Data are shown as mean ± SD. **p < 0.01, ***p < 0.001, ****p < 0.0001 (Lv-control vs Lv-PLIN2); ^##^*p* < 0.01, ^###^*p* < 0.001, ^####^*p* < 0.0001 (Lv-PLIN2 vs Lv-PLIN2 + SSO).

Based on these results, we clarified that PLIN2 promotes CRC progression in vitro which can be abolished by the CD36 antagonist SSO. To verify the oncogenic effect of PLIN2 in vivo, BALB/c nude mice were subcutaneously implanted SW480 cells to establish subcutaneous xenograft model (Fig. 8A) and CRC orthotopic model (Fig. 8B).

In the subcutaneous xenograft mouse model, we observed that the tumor volume and weight in the PLIN2 overexpression group were greater than those in the control group, whereas the tumor volume and weight in the CD36 inhibitor group was smaller than that in the PLIN2 overexpression group (Fig. 8C–E). To assess the proliferative activity of tumor cells in this model, we performed Ki67 staining of tissue sections. The expression of Ki67 was increased in the PLIN2 overexpression group compared with the control group, and this effect was reversed by treatment with the SSO (Fig. 8F, G). In addition to this, we performed IHC staining for EMT-related markers (E - cadherin, N - cadherin, vimentin and occludin). Compared with the control group, the expression of E - cadherin and occludin was decreased and the expression of vimentin and N - cadherin was increased in the PLIN2 overexpression group, and CD36 antagonist treatment reversed this effect (Fig. 8F, G).

In the CRC orthotopic model, we removed the tumor tissue on day 21 after tumor injection. We observed that the tumor weight in the PLIN2 overexpression group was greater than that in the control group, which was reversed by the CD36 inhibitor (Fig. 8H, I). HE staining results showed that compared with the control group, the cells in the PLIN2 overexpression group were tightly arranged and the nuclei were large and deeply stained, while the cells in the CD36 inhibitor group were sparsely arranged and the cellular morphology was similar to that of the control group (Fig. 8J). Consistent with the subcutaneous tumor model, to assess the proliferative activity and EMT activity in this model, we performed IHC staining of tissue sections for Ki67 and EMT-related indicators. Compared with the control group, the expression of Ki67 was increased in the PLIN2 overexpression group, the expression of E - cadherin and occludin was decreased, and the expression of vimentin and N - cadherin was increased, whereas the treatment with the CD36 antagonist reversed the above effects (Fig. 8K, L).

These results suggest that PLIN2 overexpression promotes tumor growth and EMT activity in vivo, which was reversed by treatment with the CD36 inhibitor SSO.

## Discussion

Our previous studies have focused on perioperative tumor immune dysfunction, particularly monocytes/macrophages [16, 46, 48–53]. In the present study, we constructed a new prognostic model for CRC. In this prognostic model, the PLIN2 gene presented a prognostic potential. PLIN2 acts as a wrapper around intracellular lipid droplets, maintains their stability, and participates in lipid metabolism [25]. In recent years, researchers have found that PLIN2 is associated with many types of tumors. For example, PLIN2 expression is upregulated in liver cancer [31], renal cell carcinoma [27], oral squamous carcinoma [34], and breast cancer [29] and is associated with poor prognosis. Matsubara et al. found that PLIN2 is promising as a plasma screening marker for early CRC [35]. However, the expression pattern of PLIN2 in the tumor parenchyma and the mechanism of cancer promotion are unknown. Predicted by TCGA-CRC and GEO public databases, we found that PLIN2 expression was up-regulated in CRC and correlated with poor CRC prognosis. Our tissue samples confirmed the same findings. In addition to this, PLIN2 expression was positively correlated with CRC clinical stage.

Previous studies revealed that PLIN2 is expressed in monocytes/macrophages [23, 34, 39, 40]. In the present study, we validated a specific high expression profile of PLIN2 in monocytes/macrophages subpopulations by integrating multidimensional data from spatial transcriptomics, scRNA-seq and bulk RNA-seq databases. Particularly importantly, the present study innovatively found that PLIN2 significantly promotes macrophages polarization towards the M2 type. More studies are needed in the future to show the molecular pathways by which PLIN2 regulates M2 polarization and the significance of lipid metabolic reprogramming in the immunosuppressive microenvironment.

In the study, we used two CRC cell lines, RKO and SW480 cells, to explore the effects of PLIN2 on CRC in vitro and in vivo. Overexpression of PLIN2 promoted CRC cell proliferation, migration and invasion in vitro. Conversely, knockdown of PLIN2 suppressed the proliferation, migration and invasion of CRC cells in vitro. In subcutaneous xenograft model and CRC orthotopic model, we found that overexpression of PLIN2 promoted tumor growth. These results suggest that PLIN2 promotes CRC growth and metastasis in vitro and in vivo.

EMT is an important biological process in which epithelial cells lose cell polarity and adhesion properties and acquire mesenchymal properties of enhanced migration and invasive capacity [11, 12]. This process plays a crucial role in embryonic development, wound healing and cancer progression [63]. In the context of CRC, EMT promotes tumor metastasis and is associated with a poor prognosis [8, 10, 13]. Our study used GSEA to investigate the downstream pathways regulated by the PLIN2 gene in CRC. The results showed significant enrichment of EMT-related pathways, suggesting that PLIN2 may play a key role in promoting EMT in CRC. Western blot assay showed that PLIN2 overexpression resulted in a decrease in the epithelial markers E - cadherin and occludin and an increase in the mesenchymal markers vimentin and N - cadherin in RKO and SW480 cells. Meanwhile, in subcutaneous xenograft model and CRC orthotopic model, we found the same conclusion.

The relationship between EMT and lipid metabolism is an emerging area of research. Lipid droplets are known to provide energy and biosynthetic precursors for cell proliferation [20, 42]. Recent studies have shown that lipid metabolism can affect EMT by regulating TGF-β [43, 64], AKT [65], ERK [65], CREB-Smad2/3 [66], PI3K/Akt/mTOR [67] signaling pathways. As a lipid droplet-encapsulated protein, PLIN2 may activate the EMT process in CRC by regulating lipid metabolism.

CD36, also known as fatty acid transporter protein, plays a key regulatory role in cellular lipid metabolism [41]. It has been shown that CD36-mediated endocytosis transports FAs into cells [68]. CD36-mediated fatty acid uptake leading to lipid accumulation not only provides cells with energy reserves but also has immunosuppressive effects [69]. By regulating lipid metabolism, CD36 accelerates tumor growth and metastasis, mediates chemo- and radio-resistance, and modulates the tumor immune microenvironment [70]. In several tumor types, CD36 is closely associated with EMT, such as gastric cancer [44, 67], hepatocellular carcinoma [71] and cervix cancer [43]. Besides, Hou et al. found that high glucose-induced EMT in renal tubular epithelial cells is mediated by CD36 [72]. Both as a lipid metabolism protein, there are no reports on the relationship between PLIN2 and CD36 to date. Similar to PLIN2, CD36 was also associated with the prognosis of CRC patients. Further experiments suggest that PLIN2 upregulates the expression level of CD36 and interacts with CD36. SSO is an inhibitor of CD36, inhibiting its uptake of FAs by irreversibly binding to CD36 [73]. In our study, the inhibitor of CD36, SSO, reversed the pro-oncogenic effects of PLIN2 in CRC both in vivo and in vitro. In addition to this, SSO also reversed PLIN2-induced EMT. This suggests that the effects of PLIN2 in promoting proliferation, migration, invasion and EMT in CRC cells are mediated via CD36. However, whether clinical use of CD36 inhibitors has the effect of inhibiting CRC growth and reducing metastasis Beyond this, the safety profile is unclear.

Fasting conditions (low FAs environment) exhibit potential inhibition of CRC progression. Our previous studies have shown that the expression of the cholesterol synthesis-related gene FDFT1 was upregulated under fasting conditions, thereby inhibiting CRC progression [17]. Enrichment of Bifidobacterium pseudolongum (B. pseudolongum) was observed under simulated fasting conditions, which in turn promoted the generation of memory CD8 + T cells and inhibited CRC progression [74]. The role of high FAs environment in regulating CRC should not be overlooked as well. Obesity is closely associated with poor CRC prognosis [75–77]. Epidemiological data show that obesity increases the risk of CRC by 50% and the associated mortality by 30% [78]. While the present study was conducted without FAs enrichment, a more comprehensive understanding of the PLIN2-CD36 axis may be achieved by integrating FAs-enriched models. FAs-enriched in vivo and in vitro models, or clinical analyses stratified by patient Body Mass Index (BMI), may help elucidate whether lipid overload further potentiates this axis. Such insights may ultimately support the development of precision therapies targeting lipid metabolism in obese CRC patients.

In conclusion, our study identified PLIN2 as a key prognostic biomarker in CRC and revealed its dual role in promoting tumor progression. PLIN2 facilitates macrophage polarization toward the M2 phenotype and activates the CD36-dependent EMT pathway in CRC cells, thereby enhancing tumor aggressiveness. These findings underscore the oncogenic potential of PLIN2 and suggest it may serve as a promising therapeutic target for inhibiting metastasis and improving clinical outcomes in CRC.

## Supplementary information

Supplementary Figure Legends

Figure S1

Figure S2

Figure S3

Figure S4

Figure S5

Figure S6

Figure S7

Supplementary Tables

Original Western blots

## Data Availability

The data that support the findings of this study are available from the corresponding authors upon reasonable request.
