# Supplementary material for: PLIN2 promotes colorectal cancer progression through CD36-mediated epithelial-mesenchymal transition
Source: Cell Death Dis. 2025 Jul 10;16(1):510. doi: 10.1038/s41419-025-07836-1 (PMC12246428; doi:10.1038/s41419-025-07836-1)
Supplement: Supplementary file 1 — Supplementary Figure Legends [file 41419_2025_7836_MOESM1_ESM.docx]

**Fig. S1 Schematic diagram for constructing a prognostic model for CRC.**

**Fig. S2 Screening and verifying 1204 monocytes/macrophages-related genes.**

**A** Schematic of principal component analysis of 8 immune cell datasets before (left) and after (right) removal of batch effects.

**B** Sample clustering analysis.

**C** Analysis of the network topology for various soft-thresholding powers.

**D** Gene dendrogram and module colors. The dynamic cutting algorithm obtained a total of 16 co-expression modules with different colors. The gray module was a gene set that could not be aggregated into other modules.

**E** UMAP plots for the EMTAB8170 dataset, showing cell clusters (left) and 1204 gene signatures label (right).
**F** UMAP plots for the GSE139555 dataset, showing cell clusters (left) and 1204 gene signatures label (right).
**G** UMAP plots for the GSE146771_10X dataset, showing cell clusters (left) and 1204 gene signatures label (right).
**H** UMAP plots for the GSE146771_Smartseq2 dataset, showing cell clusters (left) and 1204 gene signatures label (right).
**I** UMAP plots for the GSE166555 dataset, showing cell clusters (left) and 1204 gene signatures label (right).

**Fig. S3 Verification of a prognostic risk model based on 6 gene signatures.**

**A** Change track of each independent variable. The horizontal axis represents the log value of lambda, and the vertical axis represents the coefficient of the independent variable.

**B** The risk score, survival time, survival status, and expression of the 6 gene signatures in the TCGA-CRC cohort.

**C** Multivariate-Cox regression analysis of risk model in the TCGA-CRC cohort.

**D** Construction of the nomogram predicting survival for CRC patients in the TCGA-CRC cohort.

**E-F** Kaplan - Meier method (E) and ROC analysis (F) comparing survival of patients with high and low risk score scores in the TCGA dataset (n=594, 10-fold cross-validation).

**G-H** Kaplan - Meier method (G) and ROC analysis (H) comparing survival of patients with high and low risk score scores in the GSE17536 dataset (n=177).

**I-J** Kaplan - Meier method (I) and ROC analysis (J) comparing survival of patients with high and low risk score scores in the GSE17538 dataset (n=232).

**K-L** Kaplan - Meier method (K) and ROC analysis (L) comparing survival of patients with high and low risk score scores in the GSE36582 dataset (n=577).

**Fig. S4 Integration of scRNA-seq and Bulk RNA-seq datasets to validate the expression distribution and prognostic potential of PLIN2.**

**A** UMAP plots for the EMTAB8170 dataset, showing cell clusters (left) and PLIN2 expression (right).
**B** UMAP plots for the GSE139555 dataset, showing cell clusters (left) and PLIN2 expression (right).

**C** UMAP plots for the GSE146771_10X dataset, showing cell clusters (left) and PLIN2 expression (right).
**D** UMAP plots for the GSE146771_Smartseq2 dataset, showing cell clusters (left) and PLIN2 expression (right).
**E** UMAP plots for the GSE166555 dataset, showing cell clusters (left) and PLIN2 expression (right).

**F** Venn diagram illustrating 685 co-expressed genes shared across five scRNA-seq datasets.

**G** Forest plot showing the univariate survival analysis of the 685 intersection genes from (F), identifying those significantly associated with CRC prognosis.

**H** Venn diagram summarizing the genes that are common to bulk RNA-seq and scRNA-seq datasets.

**I** ROC analysis comparing survival of patients with high and low PLIN2 expressions in the TCGA dataset (n=594, 10-fold cross-validation).

**J** ROC analysis comparing survival of patients with high and low PLIN2 expressions in the GSE17536 dataset (n=177).

**K** ROC analysis comparing survival of patients with high and low PLIN2 expressions in the GSE17538 dataset (n=232).

**L** ROC analysis comparing survival of patients with high and low PLIN2 expressions in the GSE36582 dataset (n=577).

**Fig. S5 PLIN2 knockdown inhibited the proliferation, migration and invasion of CRC cells in vitro.**

**A** Western blot analysis of the expression of PLIN2 after treatment with si-NC and PLIN2 siRNA in RKO and SW480 cells.

**B** CCK8 assay was used to assess cell proliferation.

**C−D** Wound-healing assay was used to assay cell migration.

**E−F** Cell migration was determined by using transwell migration assays.

**G−H** Cell invasion was determined by using transwell invasion assays. Cells invading through uncoated inserts and Matrigel-coated inserts were stained.

Data are shown as mean ± SD. * p < 0.05, ** p < 0.01, *** p < 0.001, **** p < 0.0001 (si-NC vs siPLIN2-2); ^#^ p < 0.05, ^##^ p < 0.01, ^###^ p < 0.001, ^####^ p < 0.0001 (si-NC vs siPLIN2-3)

**Fig. S6 TCGA data reveal that PLIN2 is associated with the EMT pathway in CRC.**

**A** In the TCGA dataset, the gene set of the PLIN2 high expression group was enriched in the transcriptional profiles of the tumors compared to the low expression group. The NES values for each pathway using the Hallmark gene set are shown. Functional annotation of predicted PLIN2 positive and negative expression in CRC.

**B** Positive correlation between EMT and PLIN2 expression was shown using the TCGA database of CRC (n = 596).

**Fig. S7 PLIN2 promoted CD36 stabilization by inhibiting the proteasome degradation pathway.**

**A** The Kaplan - Meier method for comparing the survival of patients with high and low CD36 expression in the GSE39582 dataset.

**B-E** Western blotting analysis showed that expression of CD36 in PLIN2-overexpressing CRC cells treated with CHX at the indicated time points.

**F-H** Western blotting analysis showed that expression of CD36 in PLIN2-overexpressing CRC cells treated with MG132.

**I-K** Western blotting analysis showed that expression of CD36 in PLIN2-overexpressing CRC cells treated with CQ.

Data are shown as mean ± SD. * p < 0.05, ** p < 0.01, **** p < 0.0001
