# Supplementary figures and images for: PLIN2 promotes colorectal cancer progression through CD36-mediated epithelial-mesenchymal transition
Source: Cell Death Dis. 2025 Jul 10;16(1):510. doi: 10.1038/s41419-025-07836-1 (PMC12246428; doi:10.1038/s41419-025-07836-1)

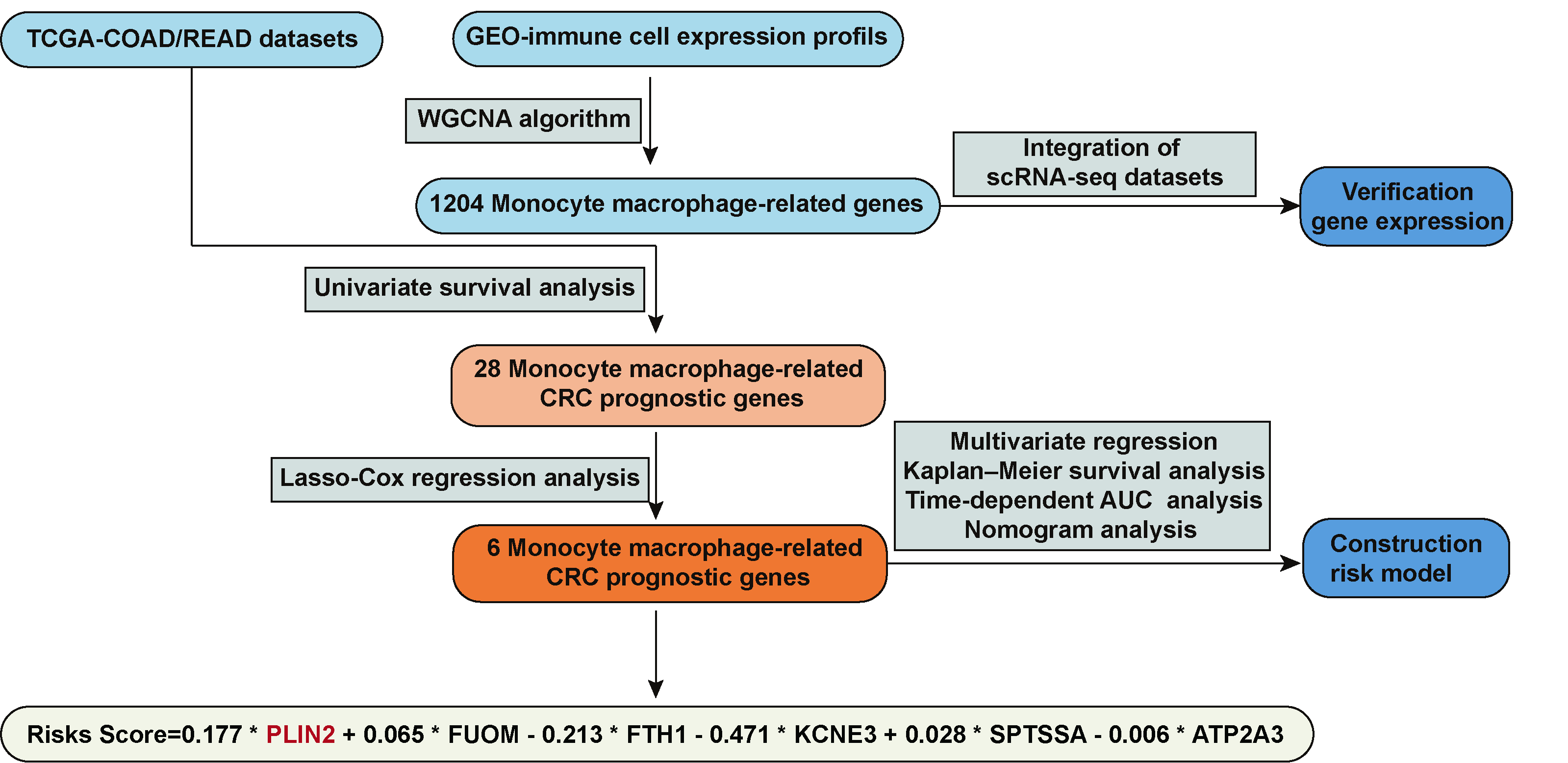

Supplement: Supplementary file 2 — Figure S1 [file 41419_2025_7836_MOESM2_ESM.tif]

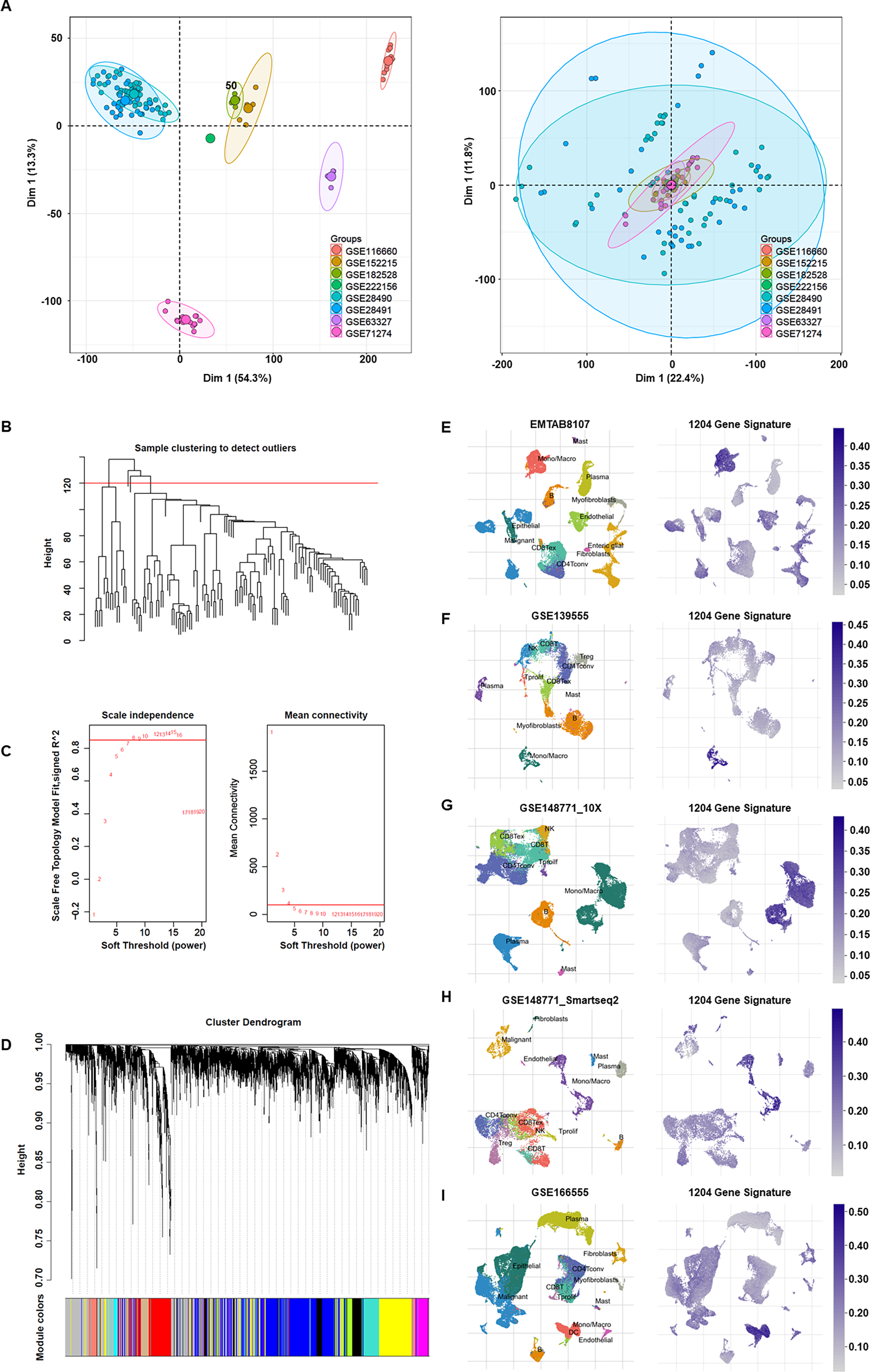

Supplement: Supplementary file 3 — Figure S2 [file 41419_2025_7836_MOESM3_ESM.tif]

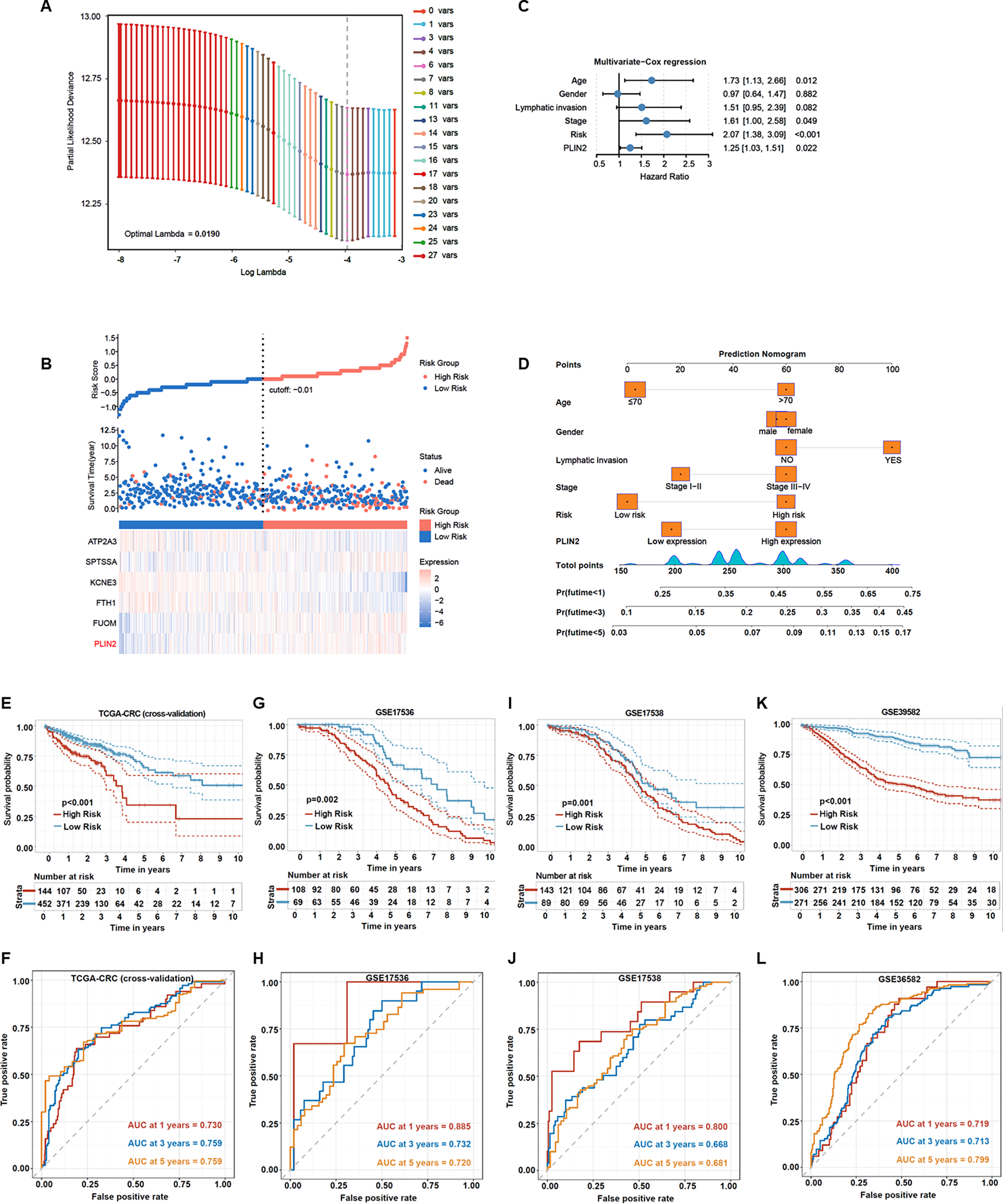

Supplement: Supplementary file 4 — Figure S3 [file 41419_2025_7836_MOESM4_ESM.tif]

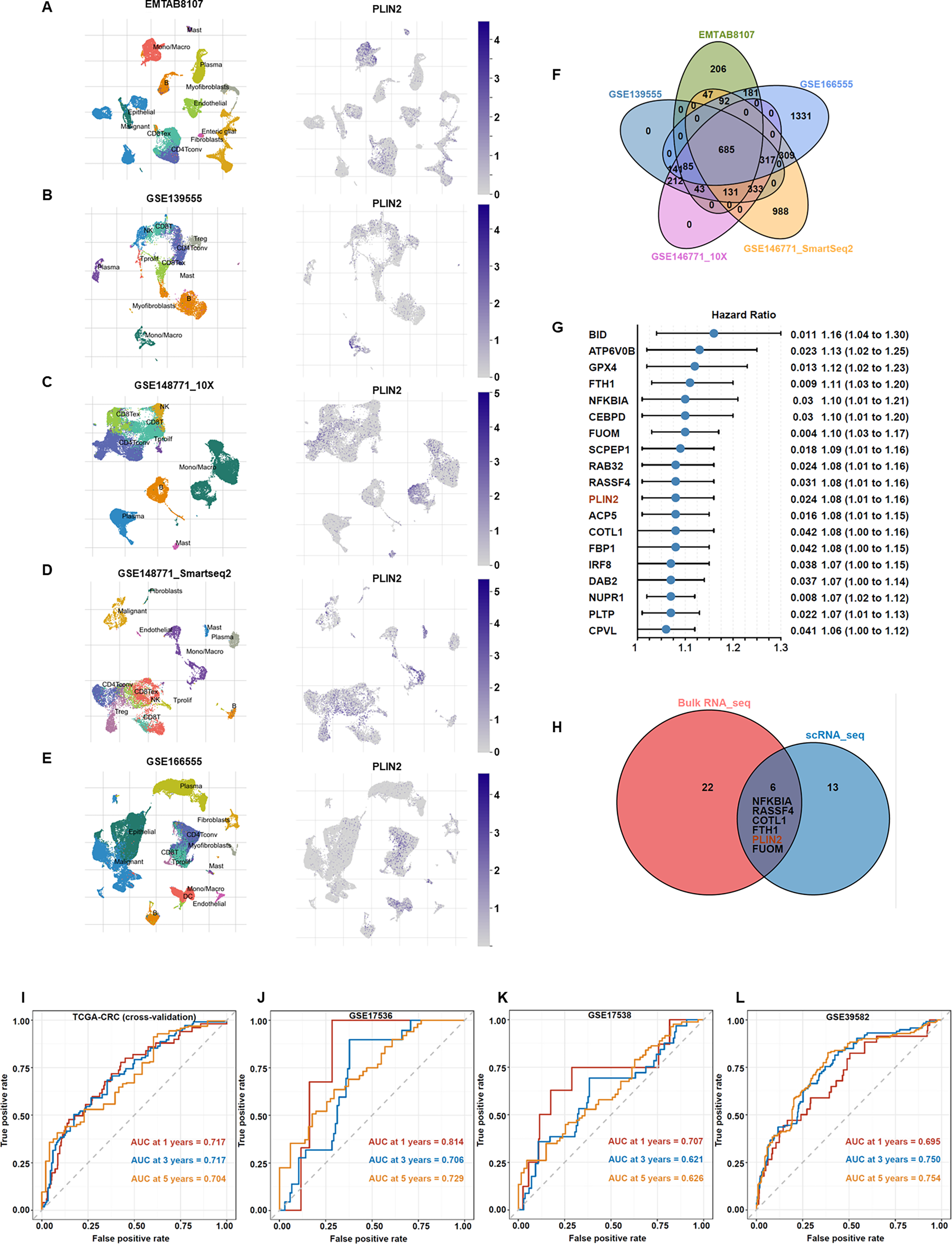

Supplement: Supplementary file 5 — Figure S4 [file 41419_2025_7836_MOESM5_ESM.tif]

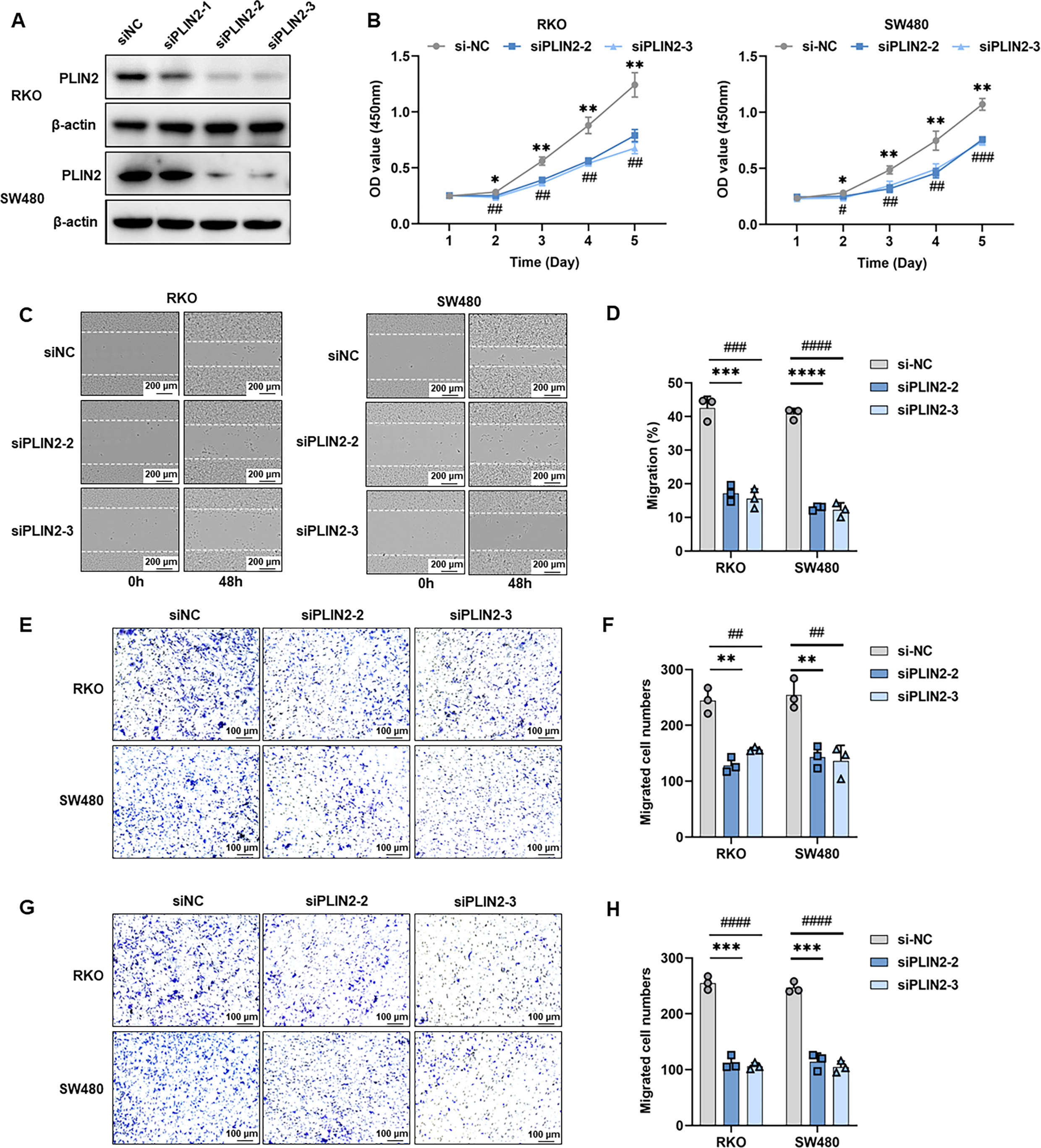

Supplement: Supplementary file 6 — Figure S5 [file 41419_2025_7836_MOESM6_ESM.tif]

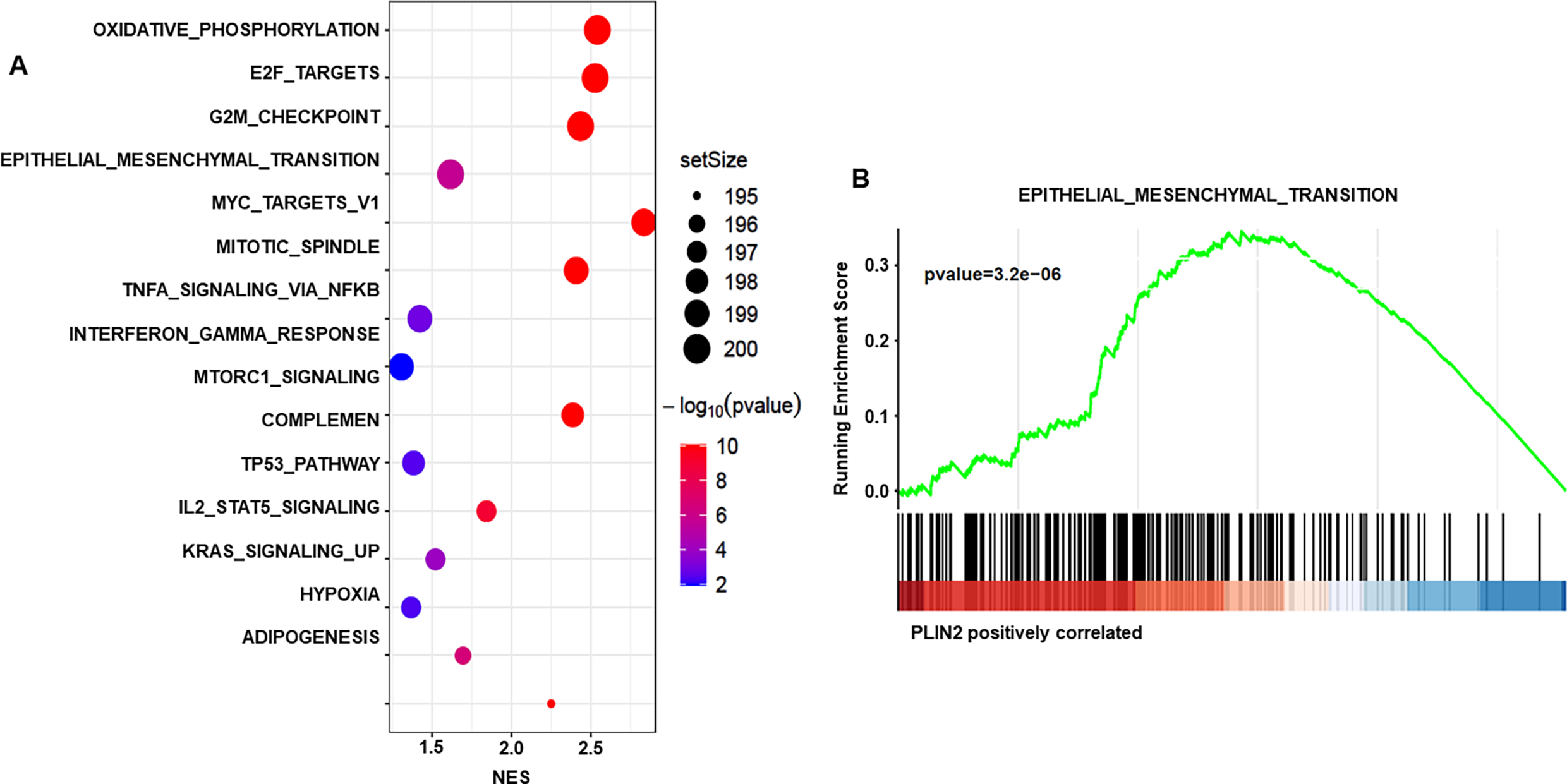

Supplement: Supplementary file 7 — Figure S6 [file 41419_2025_7836_MOESM7_ESM.tif]

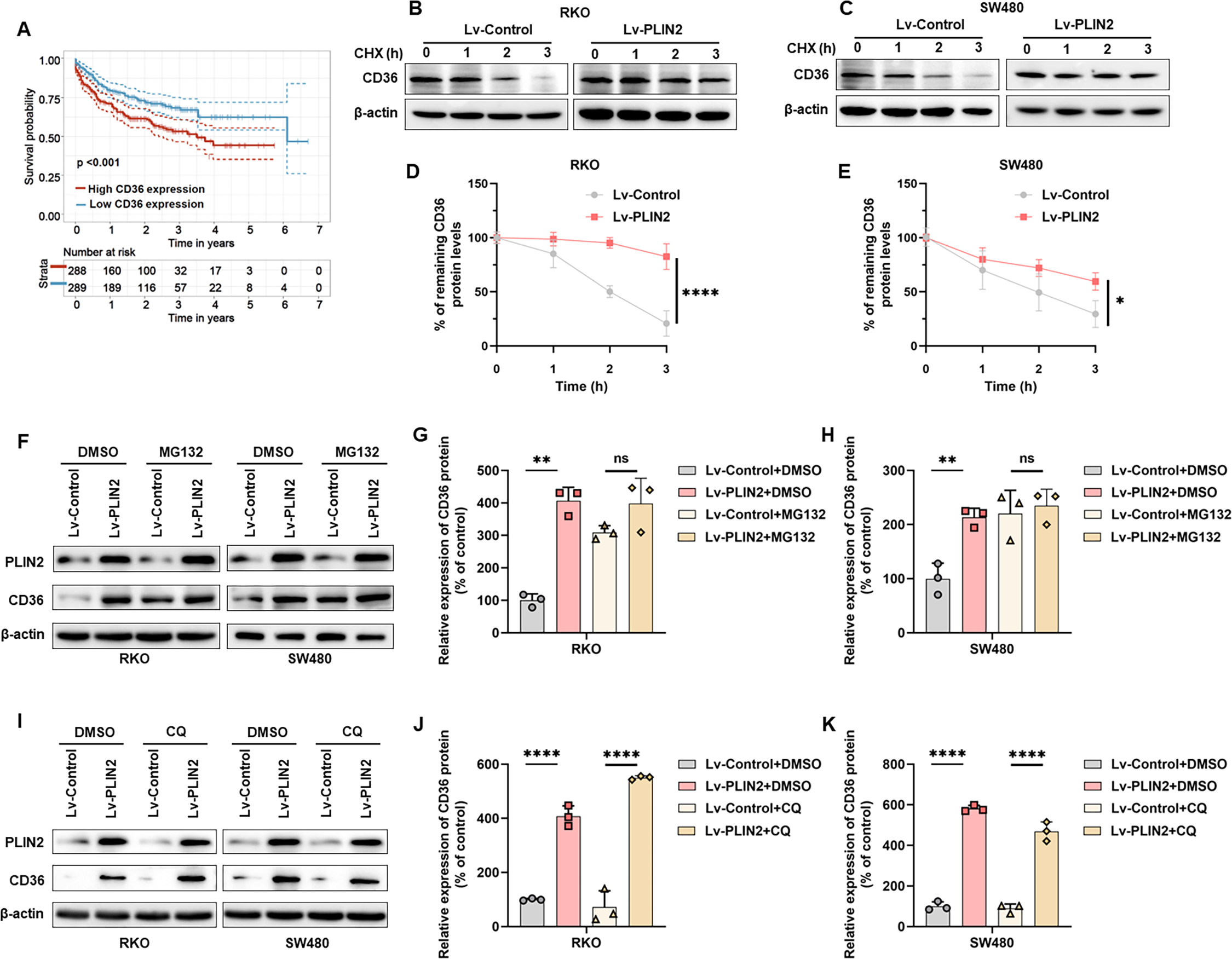

Supplement: Supplementary file 8 — Figure S7 [file 41419_2025_7836_MOESM8_ESM.tif]

Figure 4A

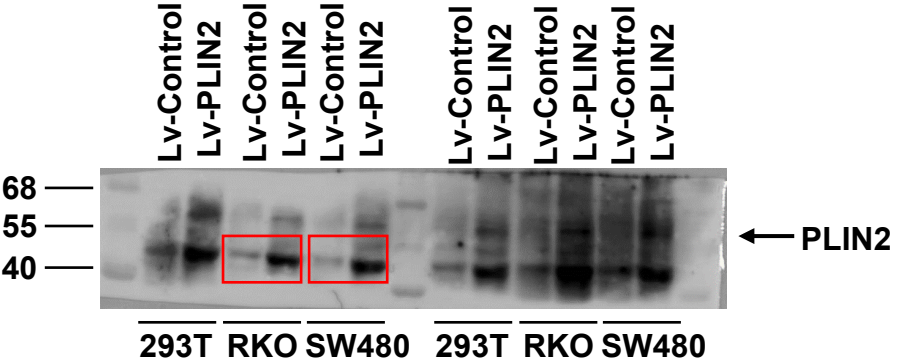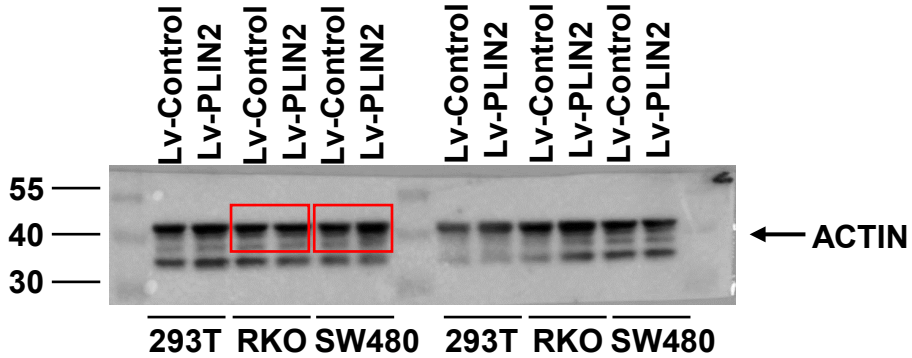

Figure 4I

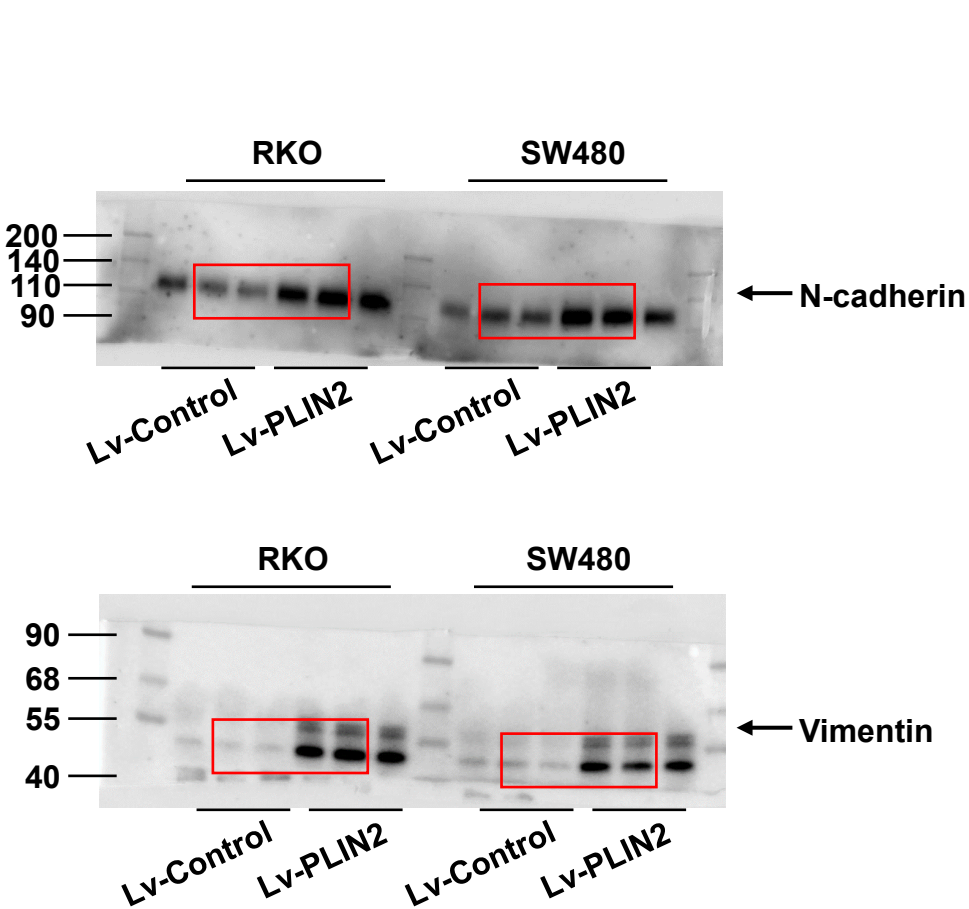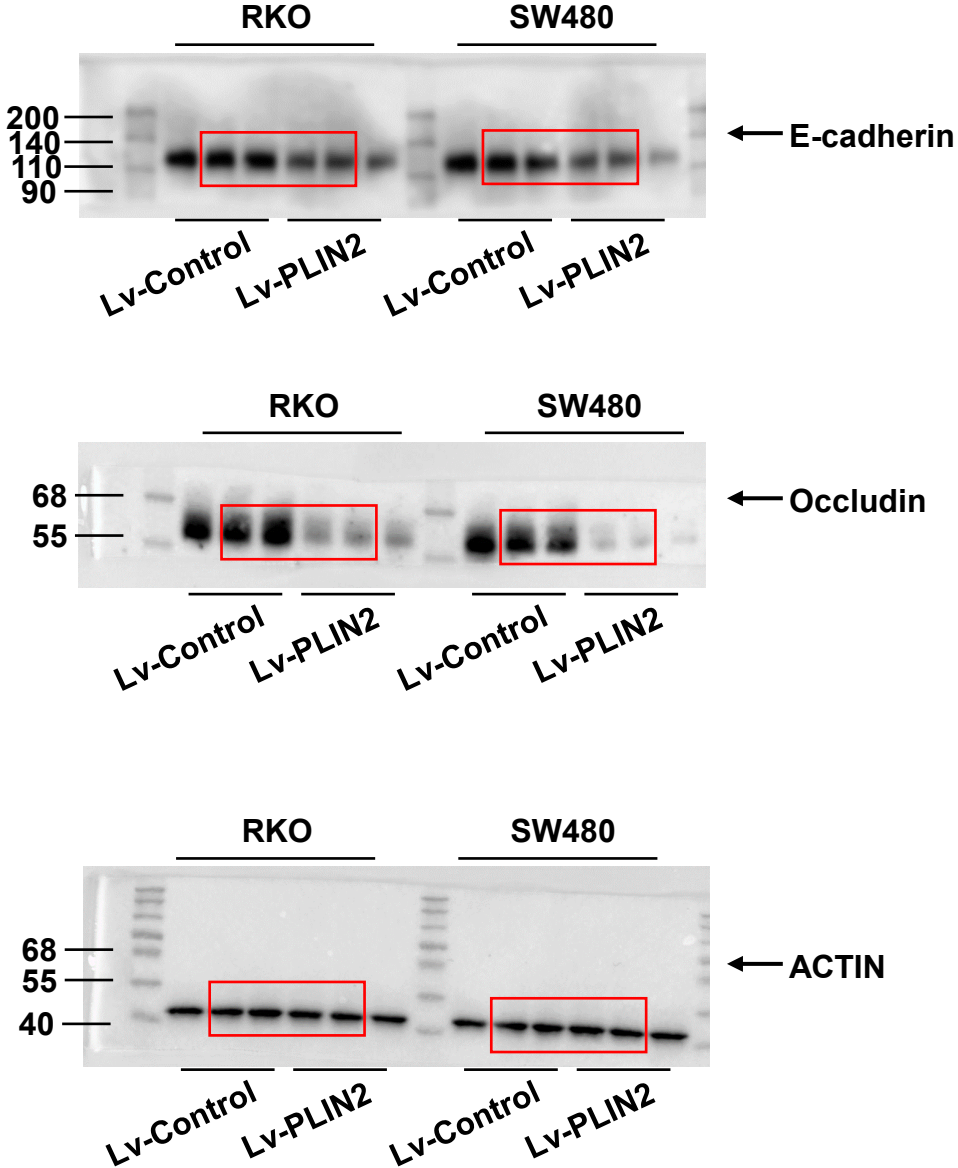

Figure 5A

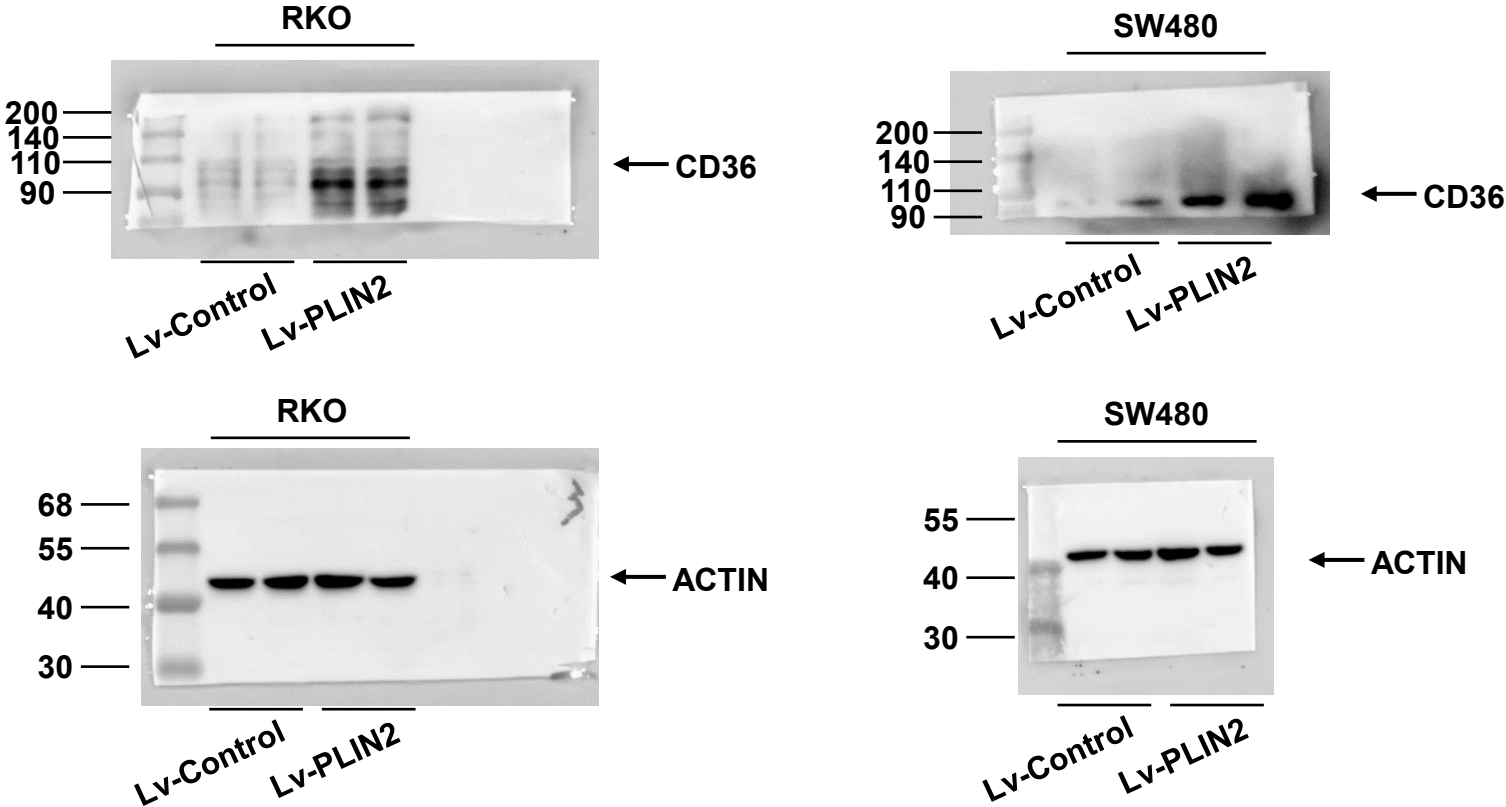

Figure 5F

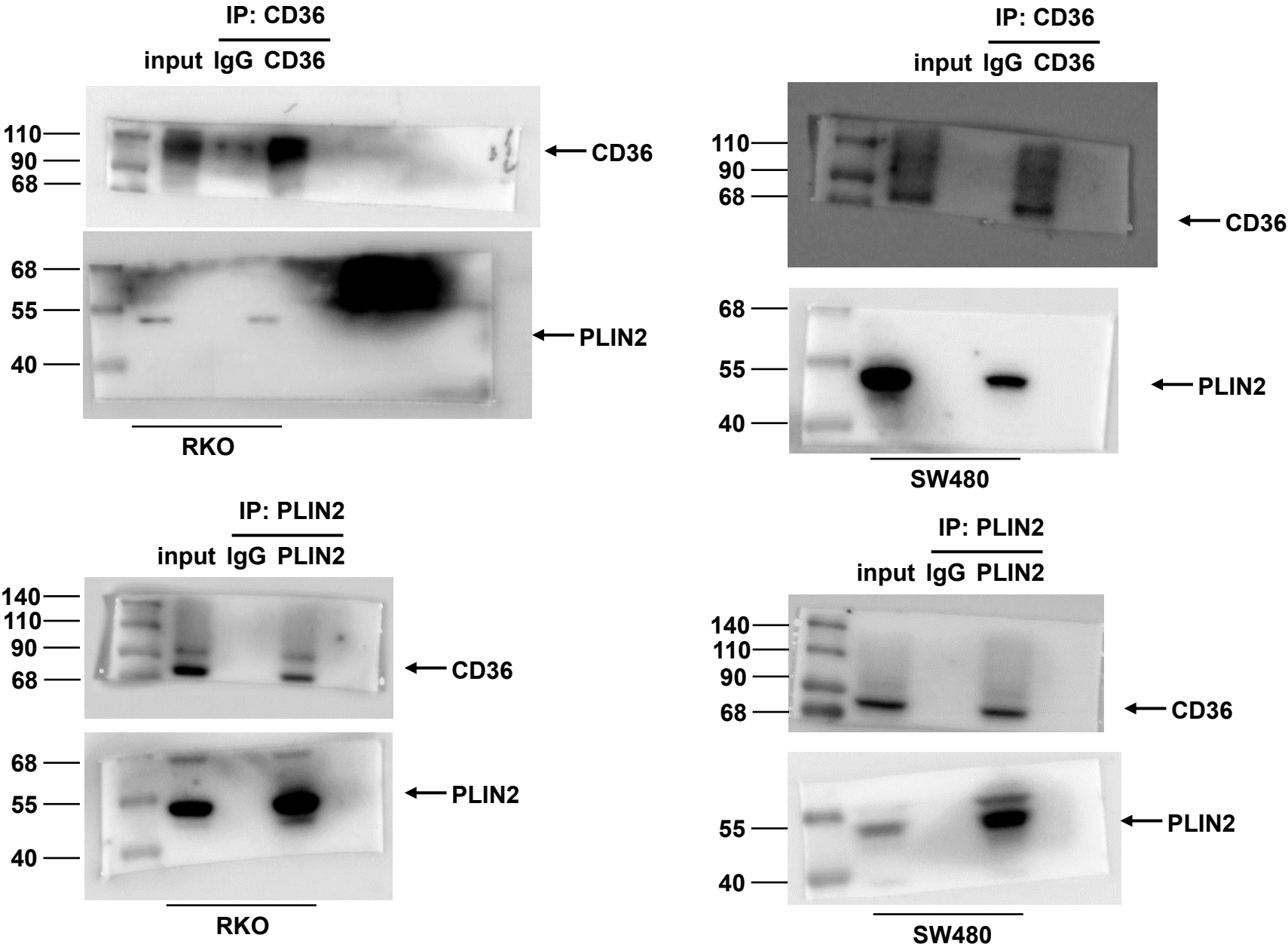

Figure 7A

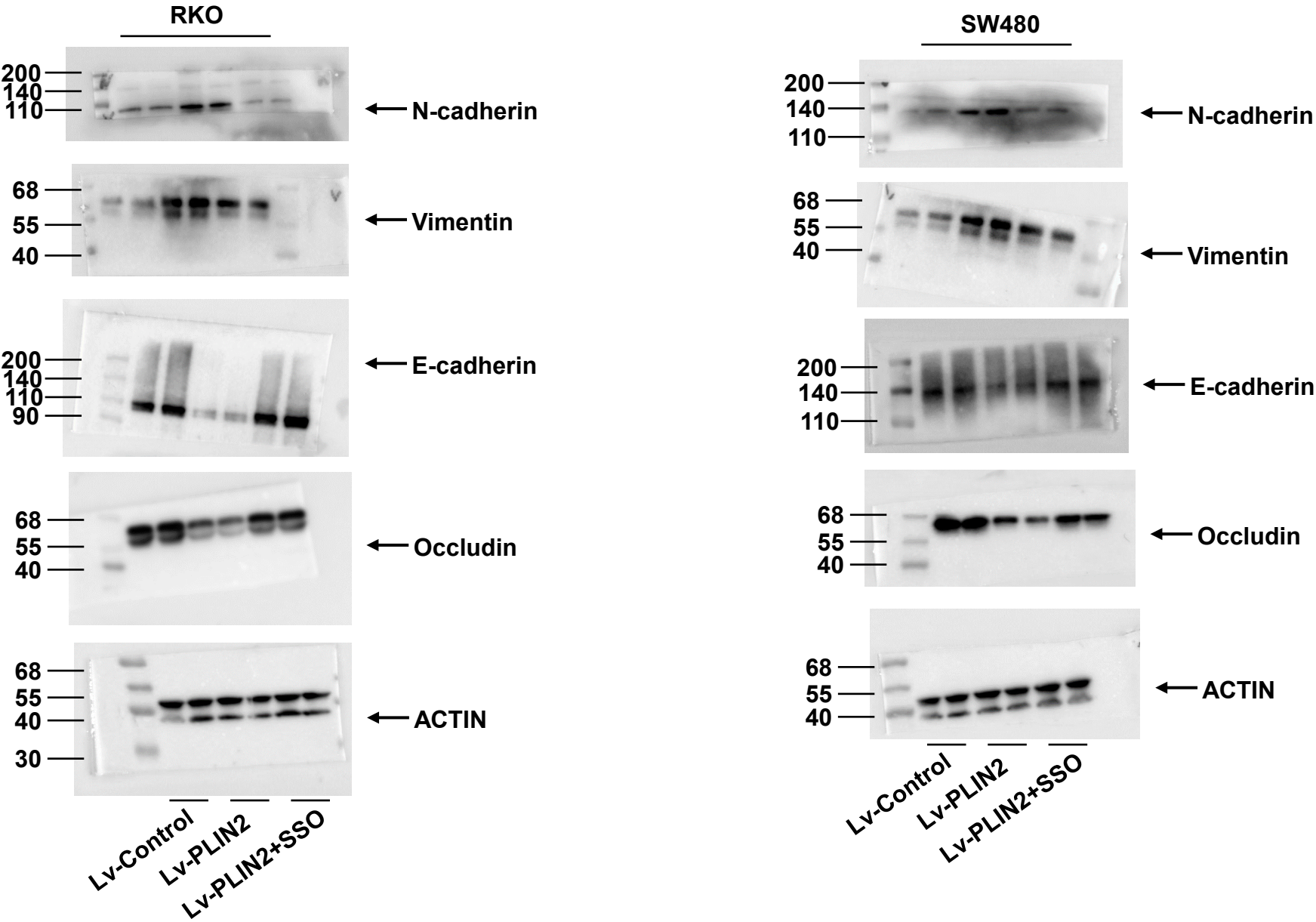

Figure S5A

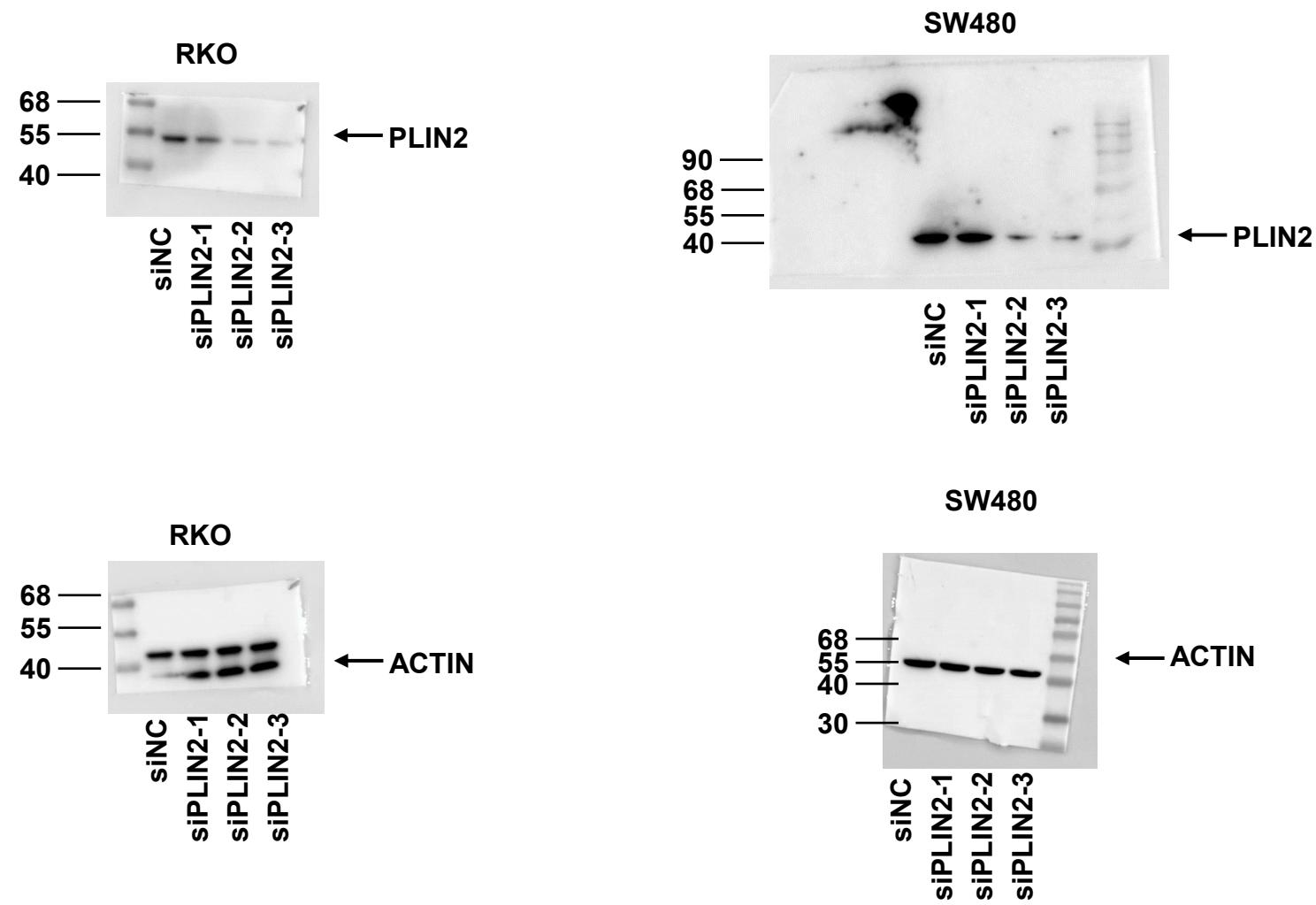

Figure S7B

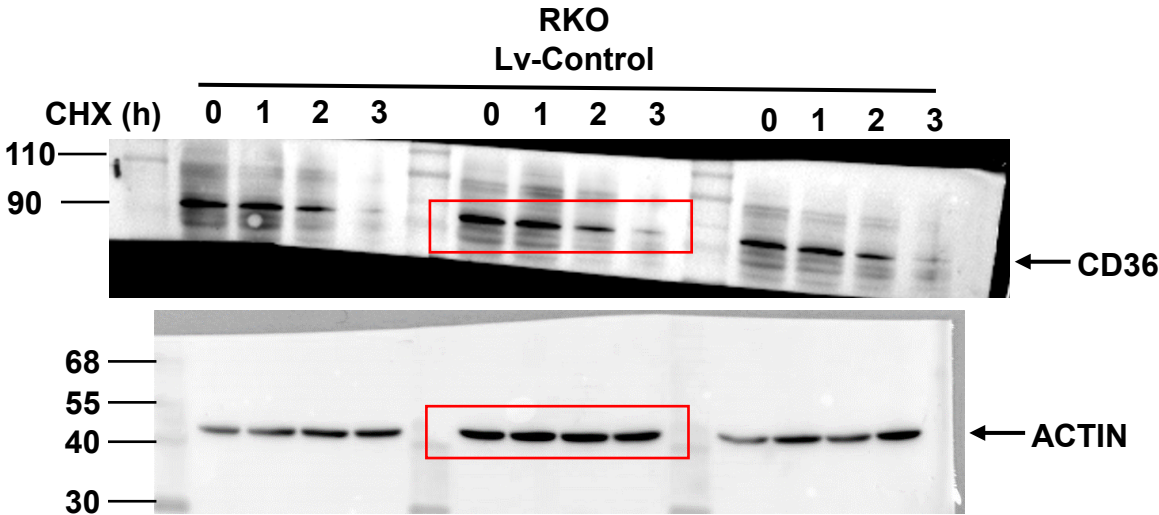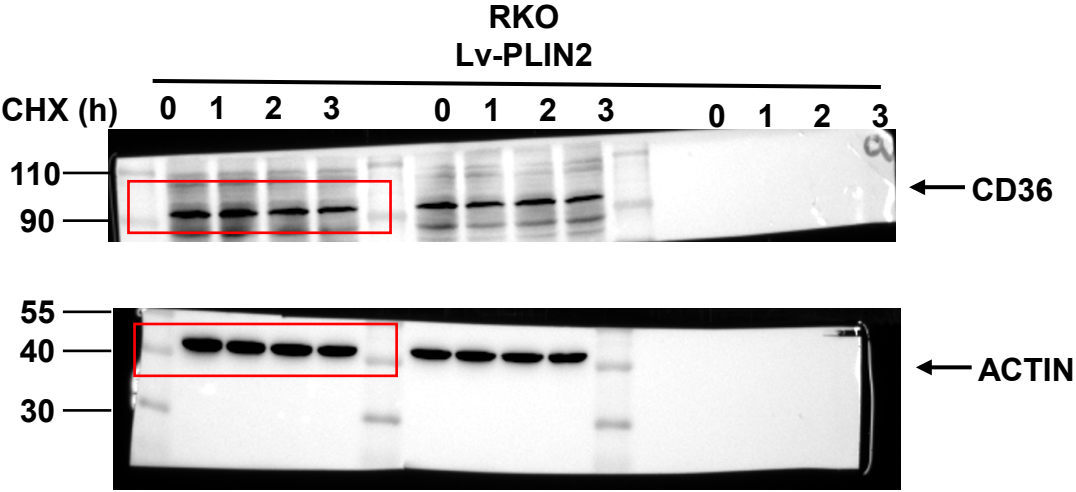

Figure S7C

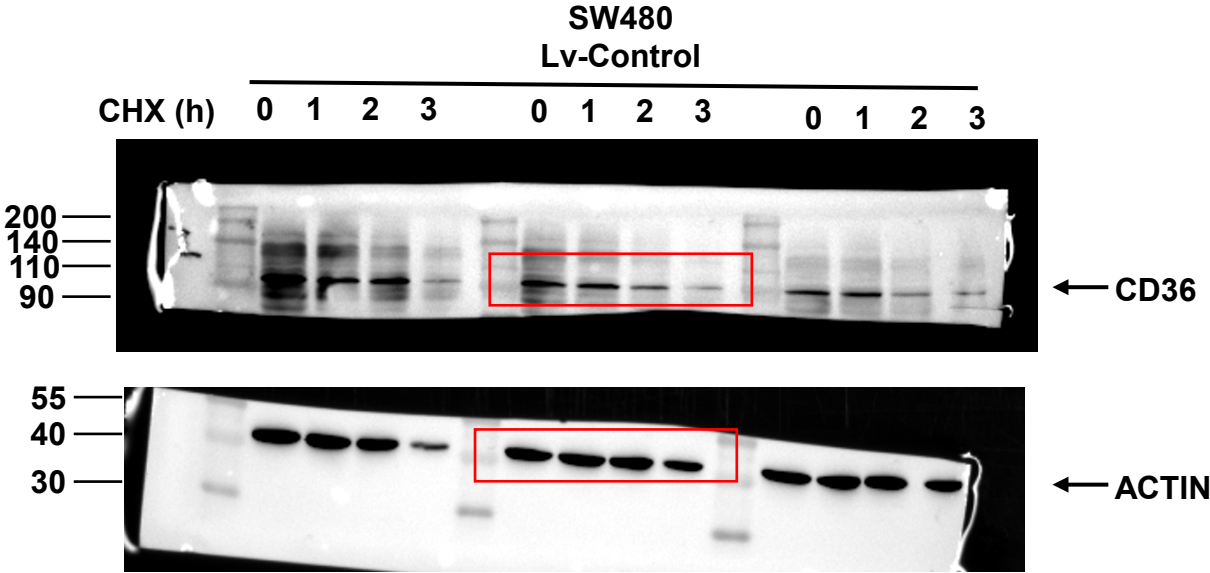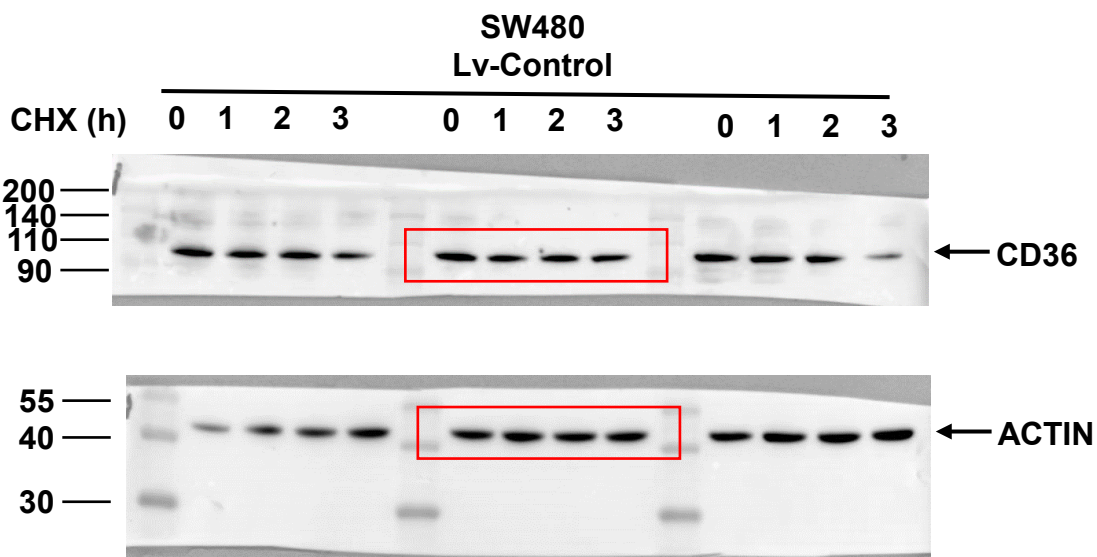

Figure S7F

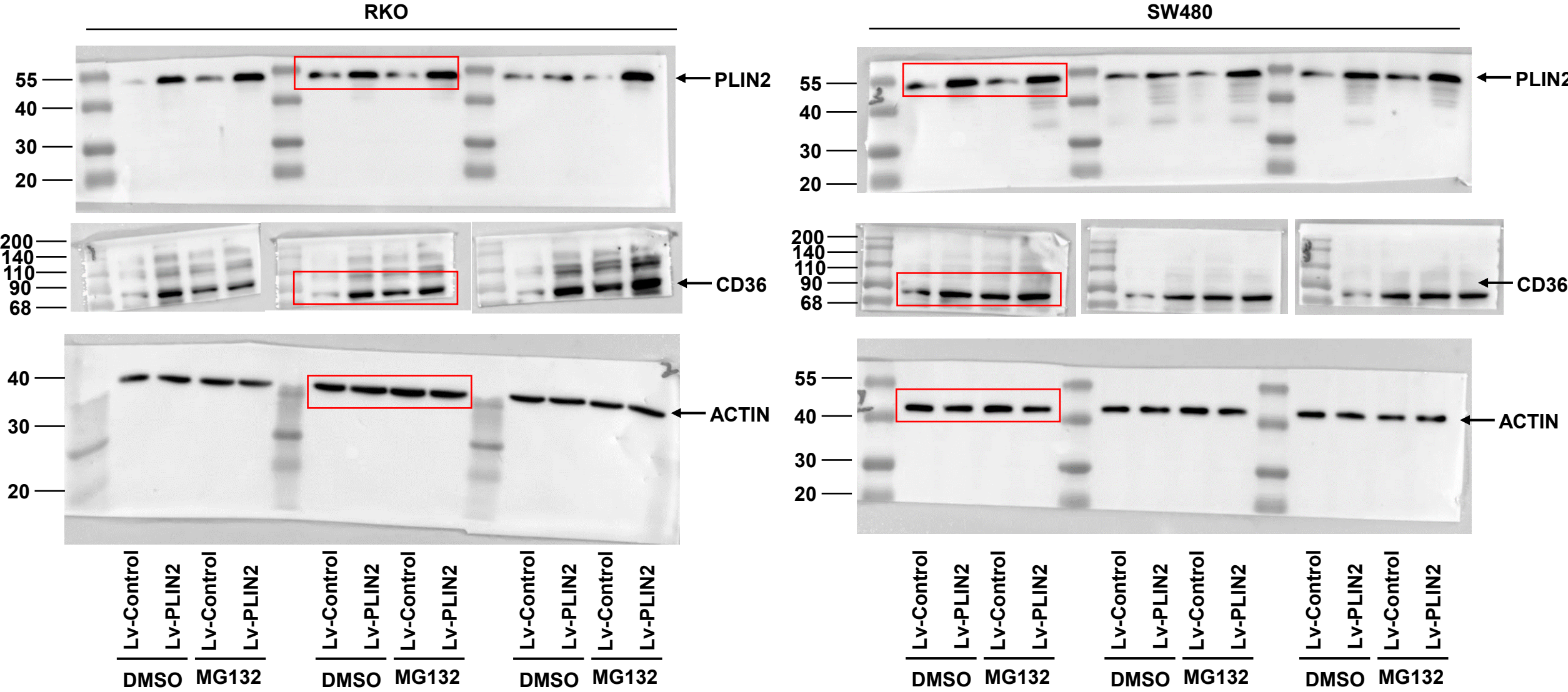

Figure S7I

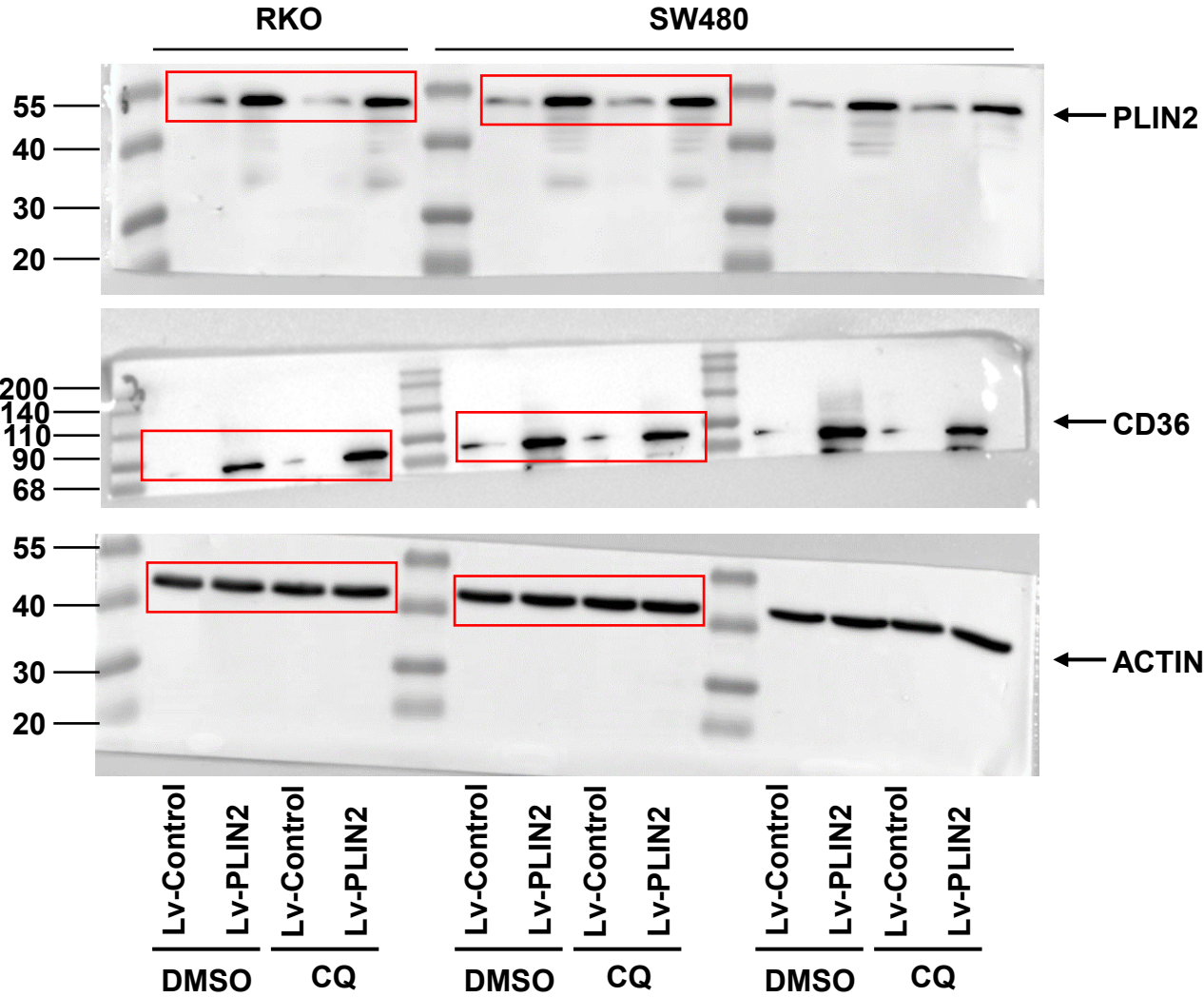

Supplement: Supplementary file 10 — Original Western blots [file 41419_2025_7836_MOESM10_ESM.pdf]
