# Supplementary material for: PLIN2 promotes colorectal cancer progression through CD36-mediated epithelial-mesenchymal transition
Source: Cell Death Dis. 2025 Jul 10;16(1):510. doi: 10.1038/s41419-025-07836-1 (PMC12246428; doi:10.1038/s41419-025-07836-1)
Supplement: Supplementary file 9 — Supplementary Tables [file 41419_2025_7836_MOESM9_ESM.docx]

**Supplemental Table S1** Primers used for qRT- PCR.

| Genes | Forward | Reverse |
| --- | --- | --- |
| CD36 | GCCAAGGAAAATGTAACCCAGG | GCCTCTGTTCCAACTGATAGTGA |
| CD86 | CCATCAGCTTGTCTGTTTCATTCC | GCTGTAATCCAAGGAATGTGGTC |
| TNF-α | CTCTTCTGCCTGCTGCACTTTG | ATGGGCTACAGGCTTGTCACTC |
| CD163 | CCAGAAGGAACTTGTAGCCACAG | CAGGCACCAAGCGTTTTGAGCT |
| CD206 | AGCCAACACCAGCTCCTCAAGA | CAAAACGCTCGCGCATTGTCCA |
| GAPDH | GGGAGCCAAAAGGGTCATCA | TGATGGCATGGACTGTGGTC |

**Supplemental Table S2** The list of antibodies.

| Protein name | Application | Dilution | Company | Catalogue number |
| --- | --- | --- | --- | --- |
| PLIN2  CD36  E-cadherin  N-cadherin  Vimentin  Occludin  β-actin  PLIN2  Ki67  E-cadherin  N-cadherin  Vimentin  Occludin  PLIN2  CD36  E-cadherin  Vimentin  PLIN2  CD36  CD86  CD206 | WB  WB  WB  WB  WB  WB  WB  IHC  IHC  IHC  IHC  IHC  IHC  IF  IF  IF  IF  Co-IP  Co-IP  FC  FC | 1/10000  1/1000  1/50000  1/10000  1/10000  1/30000  1/100000  1/200  1/5000  1/10000  1/10000  1/10000  1/10000  1/500  1/100  1/500  1/500  2 ug/ml lysate  2 ug/ml lysate  5ul/10^6^ cells  5ul/10^6^ cells | ProteinTech  Zenbio  ProteinTech  ProteinTech  ProteinTech  ProteinTech  ProteinTech  ProteinTech  ProteinTech  ProteinTech  ProteinTech  ProteinTech  ProteinTech  ProteinTech  Zenbio  ProteinTech  ProteinTech  Santa Cruz  Santa Cruz Biolegend  Biolegend | 15294-1-AP  381350  20874-1-AP  22018-1-AP  10366-1-AP  27260-1-AP  66009-1-Ig  15294-1-AP  27309-1-AP  20874-1-AP  22018-1-AP  10366-1-AP  27260-1-AP  15294-1-AP  381350  20874-1-AP  10366-1-AP  sc-377429  sc-7309  374210  321110 |

**Supplemental Table S3** Immune cell datasets obtained from GEO database.

|  | B cells | CD4+ T cells | CD8+ T cells | Treg | Eosinophils | Monocytes | NK cells | Neutrophils | Myeloid DCs | Plasmacytoid DCs | MDSCs | Total |
| --- | --- | --- | --- | --- | --- | --- | --- | --- | --- | --- | --- | --- |
| GSE71274 | 0 | 0 | 0 | 16 | 0 | 0 | 0 | 0 | 0 | 0 | 0 | 16 |
| GSE63327 | 0 | 0 | 0 | 4 | 0 | 0 | 0 | 0 | 0 | 0 | 0 | 4 |
| GSE28491 | 5 | 5 | 5 | 0 | 3 | 5 | 0 | 5 | 0 | 0 | 0 | 28 |
| GSE28490 | 5 | 5 | 5 | 0 | 4 | 10 | 5 | 3 | 5 | 5 | 0 | 47 |
| GSE222156 | 0 | 0 | 0 | 0 | 0 | 0 | 0 | 3 | 0 | 0 | 0 | 3 |
| GSE152215 | 5 | 0 | 0 | 0 | 0 | 0 | 0 | 0 | 0 | 0 | 0 | 5 |
| GSE116660 | 0 | 0 | 0 | 0 | 0 | 0 | 12 | 0 | 0 | 0 | 0 | 12 |
| GSE182528 | 0 | 0 | 0 | 0 | 0 | 0 | 0 | 0 | 0 | 0 | 5 | 5 |
| Toal | 15 | 10 | 10 | 20 | 7 | 15 | 17 | 11 | 5 | 5 | 5 | 120 |

**Supplemental Table S4** 1204 Monocytes/macrophages-associated genes.

| \| ZYX \| SCAMP2 \| MCEMP1 \| DPP4 \| \| --- \| --- \| --- \| --- \| \| ZYG11B \| SCAF11 \| MBOAT7 \| DPH2 \| \| ZSWIM6 \| SBF2 \| MBOAT2 \| DPEP2 \| \| ZNRF1 \| SAT2 \| MBOAT1 \| DPCD \| \| ZNF93 \| SAT1 \| MBD6 \| DOK1 \| \| ZNF827 \| SAAL1 \| MARVELD1 \| DOCK4 \| \| ZNF77 \| S100A9 \| MARK4 \| DNASE1L1 \| \| ZNF767P \| S100A8 \| MAPKAPK3 \| DNAL1 \| \| ZNF765 \| S100A6 \| MAPK7 \| DNAJB12 \| \| ZNF703 \| S100A12 \| MAP9 \| DNAAF9 \| \| ZNF678 \| S100A10 \| MAP4K1 \| DMXL2 \| \| ZNF671 \| RXRA \| MAP3K7CL \| DIPK1A \| \| ZNF629 \| RTTN \| MAP3K5 \| DIP2C \| \| ZNF582-DT \| RTN4 \| MAP3K3 \| DIAPH2 \| \| ZNF571 \| RTN1 \| MAP3K20 \| DHX57 \| \| ZNF550 \| RTF2 \| MAP3K11 \| DHRS9 \| \| ZNF503 \| RRP12 \| MAN2C1 \| DHRS7B \| \| ZNF500 \| RRN3P2 \| MAN2B1 \| DGCR2 \| \| ZNF493 \| RRAS \| MAN2A2 \| DESI1 \| \| ZNF467 \| RRAGD \| MAML3 \| DENND6B \| \| ZNF444 \| RPS6KA1 \| MAGED1 \| DENND5A \| \| ZNF438 \| RPL28 \| MAFG \| DENND3 \| \| ZNF354C \| RPH3A \| MAFB \| DENND1A \| \| ZNF333 \| RPGRIP1 \| MACROH2A1 \| DELE1 \| \| ZNF316 \| ROGDI \| MACF1 \| DDX5 \| \| ZNF26 \| RNPEPL1 \| LZTFL1 \| DDX41 \| \| ZNF213 \| RNPEP \| LYZ \| DDX31 \| \| ZNF185 \| RNH1 \| LYST \| DDX17 \| \| ZNF106 \| RNF40 \| LTBP1 \| DCAF12 \| \| ZMIZ1 \| RNF24 \| LTB4R \| DBNL \| \| ZFYVE21 \| RNF19B \| LST1 \| DAGLB \| \| ZFTA \| RNF149 \| LSS \| CYSTM1 \| \| ZFP30 \| RNF144B \| LSP1 \| CYRIB \| \| ZFHX3 \| RNF130 \| LRRFIP2 \| CYP1B1 \| \| ZFAND5 \| RNASE4 \| LRRFIP1 \| CYFIP2 \| \| ZFAND3 \| RNASE2 \| LRRC8D \| CYFIP1 \| \| ZEB2 \| RIPK2 \| LRPAP1 \| CYB5B \| \| ZEB1 \| RIN3 \| LRMDA \| CXCL8 \| \| ZDHHC7 \| RIN2 \| LPCAT3 \| CXCL16 \| \| ZCCHC24 \| RIN1 \| LPCAT2 \| CTSS \| \| ZCCHC18 \| RILPL2 \| LOXL3 \| CTSL \| \| ZC2HC1A \| RICTOR \| LOC202181 \| CTSD \| \| ZBTB7B \| RHOU \| LOC155060 \| CTSC \| \| ZBTB5 \| RHOH \| LOC105379362 \| CTSB \| \| ZBTB20 \| RHOG \| LOC100310756 \| CTSA \| \| YWHAG \| RHOB \| LMO2 \| CTPS1 \| \| YWHAE \| RGS18 \| LMAN1 \| CTNNA1 \| \| YPEL2 \| RGS14 \| LIPA \| CTDSP1 \| \| YPEL1 \| RGS1 \| LINC02035 \| CSTA \| \| YIF1B \| RGMB \| LINC00528 \| CST3 \| \| WEE1 \| RGL1 \| LILRA5 \| CSRP1 \| \| WDR91 \| RFX2 \| LILRA2 \| CSF3R \| \| WDR48 \| RFX1 \| LGALS3 \| CSF2RB \| \| WBP2 \| REM2 \| LGALS12 \| CSF2RA \| \| WARS1 \| RELL2 \| LGALS1 \| CSF1R \| \| VSIR \| REEP4 \| LDOC1 \| CRYZ \| \| VPS37C \| RCOR1 \| LDLRAD3 \| CRYL1 \| \| VNN3P \| RCL1 \| LCP1 \| CRTAP \| \| VNN2 \| RCAN1 \| LAT2 \| CRISPLD2 \| \| VNN1 \| RBPJ \| LASP1 \| CREB5 \| \| VIM \| RBMS2 \| LAMB2P1 \| CRAT \| \| VEGFA \| RBM47 \| LACTB \| CPVL \| \| VDR \| RBBP7 \| KSR1 \| CPT1A \| \| VCL \| RASSF4 \| KRT10-AS1 \| CPQ \| \| VCAN \| RARA-AS1 \| KRR1 \| CPPED1 \| \| VASP \| RARA \| KNTC1 \| CPNE8 \| \| USP53 \| RAP2B \| KNSTRN \| CPNE2 \| \| USP3 \| RAD17 \| KLHL8 \| CPM \| \| USP13 \| RAD1 \| KLHL3 \| CPEB2 \| \| USP11 \| RAB7A \| KLHDC8B \| CPD \| \| USB1 \| RAB40C \| KLF4 \| COTL1 \| \| URB2 \| RAB3D \| KIFAP3 \| CORO1C \| \| UNC5CL \| RAB34 \| KIF1B \| CORO1B \| \| UBN2 \| RAB32 \| KIF13A \| COQ10A \| \| UBIAD1 \| RAB31 \| KIAA2013 \| COMT \| \| UBFD1 \| RAB24 \| KIAA0930 \| COLGALT1 \| \| UBE2R2 \| RAB20 \| KIAA0513 \| COL9A2 \| \| UBE2O \| RAB11FIP3 \| KIAA0319L \| COA7 \| \| UBE2D1 \| RAB11FIP1 \| KHDC4 \| CMTM2 \| \| UBAC1 \| QSOX1 \| KDELR1 \| CMTM1 \| \| UBA5 \| QPCT \| KCTD15 \| CMIP \| \| UBA1 \| QKI \| KCTD12 \| CLUHP3 \| \| UAP1L1 \| PYGL \| KCNK6 \| CLTC \| \| TYROBP \| PYCARD \| KCNJ2 \| CLTB \| \| TYMP \| PTX3 \| KCNJ15 \| CLPB \| \| TYK2 \| PTTG1IP \| KCNE3 \| CLN6 \| \| TXNRD2 \| PTPRN2-AS1 \| KBTBD11 \| CLIP1 \| \| TUBA1C \| PTPRM \| KATNAL1 \| CLEC7A \| \| TTYH3 \| PTPRE \| JRK \| CLEC4E \| \| TTYH2 \| PTPN12 \| JPT2 \| CLEC4A \| \| TTLL4 \| PTPDC1 \| JPT1 \| CLEC12B \| \| TST \| PTGS2 \| JDP2 \| CLEC12A \| \| TSPYL2 \| PTGS1 \| JAML \| CLEC11A \| \| TSPO \| PTAFR \| JAK2 \| CKLF \| \| TSPAN4 \| PSRC1 \| JAG1 \| CKAP4 \| \| TSHZ3 \| PSAP \| ITSN1 \| CHSY1 \| \| TSEN34 \| PRRG4 \| ITPRIPL2 \| CHST13 \| \| TSC22D4 \| PRRC2B \| ITGB7 \| CHST11 \| \| TRPS1 \| PROSER2 \| ITGAX \| CHST10 \| \| TRIQK \| PROK2 \| ITGAM \| CHP1 \| \| TRIOBP \| PRKCD \| ITFG2 \| CHD6 \| \| TRIM8 \| PRKAR2B \| ITFG1 \| CFD \| \| TRIM7 \| PRKACA \| IRS2 \| CEP97 \| \| TRIM65 \| PRELID1 \| IRF5 \| CEP43 \| \| TRIM25 \| PRDX3 \| IRF2BP2 \| CENPH \| \| TRIB1 \| PRCP \| IRAK3 \| CEBPA \| \| TREM1 \| PRAM1 \| IPP \| CEACAM4 \| \| TRAPPC10 \| PRAG1 \| INSR \| CDV3 \| \| TRAF7 \| PPT1 \| INPPL1 \| CDKN1C \| \| TPP1 \| PPP2R5B \| IMPA2 \| CDKN1A \| \| TPK1 \| PPP2R1B \| IMMP2L \| CDK5R1 \| \| TPD52L2 \| PPP1R26 \| IL4I1 \| CDK5 \| \| TPCN2 \| PPP1R16B \| IL21R \| CDC42EP4 \| \| TP53RK \| PPP1R11 \| IL1RN \| CD99L2 \| \| TP53I3 \| PPM1F \| IL1RAP \| CD93 \| \| TP53BP1 \| PPIF \| IL1R2 \| CD86 \| \| TOR4A \| PPCDC \| IL1R1 \| CD68 \| \| TOR2A \| POR \| IL1B \| CD63 \| \| TOP2B \| POP1 \| IL17RA \| CD59 \| \| TOMM40L \| POLR2L \| IL15 \| CD36 \| \| TOM1 \| POLR2E \| IGSF6 \| CD320 \| \| TOLLIP \| POLE \| IGSF22 \| CD300C \| \| TNPO2 \| POLD3 \| IFTAP \| CD151 \| \| TNFSF13B \| POGZ \| IFNGR1 \| CD14 \| \| TNFRSF8 \| POC5 \| IFIT3 \| CCR5 \| \| TNFRSF1A \| PNPO \| IFI44L \| CCR1 \| \| TNFRSF12A \| PNPLA6 \| IFI44 \| CCPG1 \| \| TNFAIP8L2 \| PNN \| IFI30 \| CCNY \| \| TNFAIP6 \| PLXNC1 \| IER5 \| CCNL1 \| \| TNFAIP2 \| PLXNB2 \| IER3 \| CCDC88A \| \| TMSB15B \| PLXDC2 \| HYAL2 \| CCDC14 \| \| TMOD2 \| PLSCR1 \| HTRA2 \| CBX5 \| \| TMEM91 \| PLPPR2 \| HSPH1 \| CBLB \| \| TMEM41A \| PLPP1 \| HSPA5 \| CATSPER1 \| \| TMEM33 \| PLOD3 \| HS1BP3 \| CATIP-AS1 \| \| TMEM250 \| PLOD1 \| HPSE \| CASTOR3P \| \| TMEM176B \| PLIN3 \| HPS4 \| CAST \| \| TMEM170B \| PLIN2 \| HOMER3 \| CASP5 \| \| TMEM164 \| PLEKHO2 \| HNRNPH1 \| CARNS1 \| \| TMEM161B \| PLEKHM1 \| HMOX1 \| CARD9 \| \| TMEM14A \| PLEK \| HMGN4 \| CARD19 \| \| TMEM143 \| PLD2 \| HMBS \| CARD16 \| \| TMEM120A \| PLCXD2 \| HM13 \| CARD11 \| \| TMEM104 \| PLBD1 \| HLX \| CAPZA2 \| \| TMED9 \| PLAUR \| HIP1 \| CAPNS1 \| \| TMED1 \| PLA2G7 \| HIF1A \| CAMKK2 \| \| TMBIM6 \| PLA2G6 \| HIBCH \| CAMK2N1 \| \| TM9SF4 \| PKM \| HGSNAT \| CAMK1 \| \| TLR8 \| PIWIL4 \| HEXB \| CALU \| \| TLR4 \| PITPNB \| HEXA \| CALML4 \| \| TLN1 \| PIP5K1C \| HEMK1 \| CAD \| \| TLK1 \| PIP4P2 \| HEBP1 \| CACNA2D4 \| \| TLE3 \| PINK1 \| HEATR5A \| CA2 \| \| TKT \| PILRA \| HDLBP \| C9orf72 \| \| TK2 \| PIK3CB \| HDHD5 \| C5AR1 \| \| TJP2 \| PIDD1 \| HDAC8 \| C4orf48 \| \| TIMP2 \| PID1 \| HCK \| C3orf62 \| \| TIMP1 \| PICALM \| HCG18 \| C3AR1 \| \| TICAM1 \| PHF21A \| HCCS \| C3 \| \| THOC1 \| PHC3 \| HBEGF \| C2orf76 \| \| THG1L \| PHC2 \| HAL \| C20orf27 \| \| THEMIS2 \| PGM2L1 \| HADH \| C1QTNF3 \| \| THBS1 \| PGK1 \| HACE1 \| C1orf54 \| \| TGOLN2 \| PFKFB4 \| HACD4 \| C1orf162 \| \| TGFBI \| PER1 \| H4C3 \| C19orf54 \| \| TFEC \| PEPD \| H1-0 \| C17orf67 \| \| TFE3 \| PELI3 \| GSTZ1 \| C15orf39 \| \| TET3 \| PELI2 \| GSTA4 \| BRMS1L \| \| TET2 \| PECAM1 \| GRN \| BRI3 \| \| TESC \| PEA15 \| GRK2 \| BPTF \| \| TENT5A \| PDRG1 \| GRINA \| BNIP3L \| \| TECPR2 \| PDLIM7 \| GRB10 \| BLVRB \| \| TDRD9 \| PDK4 \| GRAMD4 \| BLVRA \| \| TDP1 \| PDIA5 \| GPR35 \| BLTP3B \| \| TCIRG1 \| PDGFC \| GPR34 \| BLM \| \| TCF7L2 \| PCTP \| GPCPD1 \| BID \| \| TCEA1 \| PCNT \| GPATCH2 \| BEST1 \| \| TCAIM \| PCGF5 \| GPAT3 \| BCL6 \| \| TBXAS1 \| PCBP4 \| GPAM \| BCL3 \| \| TBC1D8 \| PATL1 \| GP6 \| BCL2L2 \| \| TBC1D2 \| PARP16 \| GOLPH3L \| BCL2L13 \| \| TBC1D13 \| PARP15 \| GNS \| BCL2A1 \| \| TBC1D12 \| PARL \| GNPTG \| BCKDK \| \| TBC1D10B \| PAQR7 \| GNPDA1 \| BCKDHB \| \| TAP2 \| PAQR4 \| GNA15 \| BCKDHA \| \| SYNJ1 \| PALB2 \| GLMN \| BCAT1 \| \| SYNGR1 \| PAK1 \| GLIPR1 \| BAZ2B \| \| SYNE2 \| PADI4 \| GLCCI1 \| BARD1 \| \| SUSD6 \| P2RY13 \| GLB1L \| BAIAP2-DT \| \| SUSD1 \| P2RY10 \| GLA \| BACH2 \| \| SUMF1 \| P2RX7 \| GK \| BACE1 \| \| SULF2 \| P2RX4 \| GGTA1 \| B9D2 \| \| SUFU \| OVGP1 \| GDF11 \| B3GNTL1 \| \| STXBP2 \| OSGIN2 \| GDE1 \| B3GNT5 \| \| STX4 \| OSBPL1A \| GASK1B \| AVPI1 \| \| STX3 \| OLIG1 \| GAS2L1 \| AUTS2 \| \| STX11 \| OGT \| GARS1-DT \| ATRN \| \| STX10 \| OGFRL1 \| GALNT7 \| ATPAF2 \| \| STRN4 \| OGFR \| GALNT6 \| ATP8A1 \| \| STMN1 \| ODF3B \| GAB2 \| ATP6V1B2 \| \| STK38L \| OCEL1 \| GAA \| ATP6V1A \| \| STEAP4 \| OAZ2 \| G6PD \| ATP6V0C \| \| STEAP3 \| OAS1 \| G6PC3 \| ATP6AP1 \| \| STARD9 \| OAF \| G0S2 \| ATP2B1-AS1 \| \| STARD3NL \| NUP214 \| FUOM \| ATP2A3 \| \| STAG1 \| NUP210 \| FUCA2 \| ATP1B1 \| \| ST3GAL6 \| NUMA1 \| FUCA1 \| ATP11A \| \| SSR1 \| NUDT16 \| FTH1P5 \| ATOX1 \| \| SRSF2 \| NUDT14 \| FTH1 \| ATG7 \| \| SRSF1 \| NT5C2 \| FRYL \| ATG3 \| \| SRGAP2 \| NSFL1C \| FRY \| ATG16L2 \| \| SRA1 \| NRROS \| FRMD4B \| ATF6 \| \| SPTSSA \| NRGN \| FRMD3 \| ATF5 \| \| SPNS1 \| NR4A1 \| FRAT2 \| ATF3 \| \| SPHK1 \| NQO2 \| FRAT1 \| ASRGL1 \| \| SPECC1 \| NPTN \| FPR2 \| ASL \| \| SPATS2 \| NPL \| FPR1 \| ASGR1 \| \| SPARC \| NPC2 \| FOSB \| ASB13 \| \| SPAG9 \| NOD2 \| FOS \| ASAP1-IT2 \| \| SORT1 \| NMB \| FOLR3 \| ASAP1 \| \| SOCS3 \| NLRP3 \| FMNL3 \| ASAH1 \| \| SOCS1 \| NKAPP1 \| FMNL1 \| ARSD \| \| SNX30 \| NIPSNAP3A \| FLVCR2 \| ARRDC4 \| \| SNX27 \| NINJ2 \| FLOT1 \| ARRDC1 \| \| SNX1 \| NINJ1 \| FLII \| ARRB2 \| \| SNRPN \| NIBAN2 \| FKBP1A \| ARRB1 \| \| SMYD5 \| NHS \| FKBP15 \| ARPC4 \| \| SMIM3 \| NFKBIL1 \| FIGNL1 \| ARID4B \| \| SMC4 \| NFKBIA \| FHOD1 \| ARID3A \| \| SMARCD3 \| NFIC \| FGR \| ARID2 \| \| SMARCAD1 \| NFE2 \| FGL2 \| ARHGEF40 \| \| SLC9B2 \| NFATC2 \| FGD4 \| ARHGEF2 \| \| SLC7A7 \| NFAM1 \| FFAR2 \| ARHGEF10L \| \| SLC7A6 \| NETO2 \| FEZ2 \| ARHGDIB \| \| SLC7A1 \| NEMP1 \| FES \| ARHGAP5 \| \| SLC6A6 \| NEK3 \| FERMT3 \| ARHGAP27 \| \| SLC5A6 \| NEIL2 \| FCN1 \| ARF5 \| \| SLC4A7 \| NDST1 \| FCHO2 \| ARF3 \| \| SLC4A2 \| NCOA5 \| FCER1G \| ARAP3 \| \| SLC49A4 \| NCF2 \| FCER1A \| ARAP1 \| \| SLC48A1 \| NATD1 \| FCAR \| AQP9 \| \| SLC43A3 \| NAPRT \| FBXO6 \| APLP2 \| \| SLC39A14 \| NAMPT \| FBXO4 \| APH1B \| \| SLC39A11 \| NAGPA \| FBP1 \| APAF1 \| \| SLC39A1 \| NAGA \| FBN2 \| AP5B1 \| \| SLC38A7 \| NADK \| FBF1 \| AP4B1 \| \| SLC38A1 \| NACC2 \| FAR2 \| AP3M2 \| \| SLC36A1 \| NABP1 \| FAR1 \| AP1S2 \| \| SLC31A2 \| NAAA \| FAM53B \| ANXA5 \| \| SLC30A1 \| NAA60 \| FAM50B \| ANPEP \| \| SLC27A3 \| NAA38 \| FAM32A \| ANO6 \| \| SLC27A1 \| MYPOP \| FAM234A \| ANO10 \| \| SLC26A6 \| MYOF \| FAM210B \| ANKRD46 \| \| SLC25A45 \| MYO9B \| FAM169A \| ANKRD22 \| \| SLC25A4 \| MYO7A \| FAM13A \| ANKRD12 \| \| SLC25A37 \| MYEF2 \| FAM131A \| ANAPC1 \| \| SLC25A29 \| MYDGF \| FAM110A \| AMT \| \| SLC25A17 \| MYD88 \| EXTL2 \| AMPD2 \| \| SLC25A16 \| MYADM \| EXT1 \| ALPK1 \| \| SLC25A13 \| MXD1 \| EXOSC4 \| ALG3 \| \| SLC22A4 \| MVP \| EXO5 \| ALDOA \| \| SLC22A18 \| MVB12B \| EVI2A \| ALDH3B1 \| \| SLC22A15 \| MTX1 \| EVA1B \| ALDH2 \| \| SLC19A1 \| MTMR3 \| ETV6 \| ALDH1A1 \| \| SLC16A5 \| MTF1 \| ETS2 \| ALDH18A1 \| \| SLC16A3 \| MSRB2 \| ERMAP \| AKT1 \| \| SLC15A3 \| MSRA \| ERLIN1 \| AKR1A1 \| \| SLC12A9 \| MS4A7 \| ERG28 \| AIF1 \| \| SLC12A2 \| MS4A6A \| EREG \| AHR \| \| SLC11A1 \| MRTFA \| EPM2AIP1 \| AGTRAP \| \| SLAMF6 \| MROH1 \| EPHA4 \| AGPS \| \| SIRPA \| MRAS \| EPB41L3 \| AGPAT3 \| \| SIPA1L2 \| MPST \| ENTPD1-AS1 \| AGO4 \| \| SIN3B \| MPRIP \| ENO1 \| AGAP3 \| \| SIL1 \| MPP1 \| ENG \| AFDN \| \| SIGLEC15 \| MPO \| EMP3 \| ADNP \| \| SIGLEC1 \| MPND \| EMILIN2 \| ADM \| \| SIAE \| MPDU1 \| ELL \| ADI1 \| \| SHROOM1 \| MORC4 \| ELAPOR2 \| ADGRE3 \| \| SHQ1 \| MNDA \| EIF4EBP1 \| ADGRE2 \| \| SH3PXD2A \| MITF \| EIF4A2 \| ADGRE1 \| \| SH3GL1 \| MIR23AHG \| EHD4 \| ADAP2 \| \| SH3BP2 \| MIR22HG \| EHBP1L1 \| ADAM9 \| \| SH3BGRL3 \| MIER2 \| EGR3 \| ADAM8 \| \| SH2D3C \| MICU1 \| EGR1 \| ADA2 \| \| SH2D3A \| MGST2 \| EEPD1 \| ACVR1B \| \| SFXN2 \| MGST1 \| EDC3 \| ACSS2 \| \| SETD9 \| MGAT1 \| ECHDC3 \| ACSL1 \| \| SETD3 \| MGA \| ECE1 \| ACRBP \| \| SERTAD3 \| MFSD14B \| DYSF \| ACER3 \| \| SERPINB8 \| MFSD1 \| DUT \| ACAP3 \| \| SERPINB6 \| METTL22 \| DUSP7 \| ACADVL \| \| SERPINA1 \| METTL17 \| DUSP6 \| ABTB1 \| \| SERP1 \| MERTK \| DUSP3 \| ABR \| \| SELENON \| MEGF9 \| DUSP23 \| ABHD5 \| \| SECTM1 \| MDC1 \| DUSP18 \| ABHD12 \| \| SEC24D \| MCOLN2 \| DUSP1 \| ABCA5 \| \| SDF2L1 \| MCOLN1 \| DSC2 \| ABAT \| \| SDC4 \| MCM6 \| DRAM1 \| AATBC \| \| SCO2 \| MCM2 \| DPYSL2 \| AARS1 \| \| SCAND2P \| MCL1 \| DPYD \| AAMP \| \|  \|  \|  \|  \| |  |  |  |
| --- | --- | --- | --- | --- | --- | --- | --- | --- | --- | --- | --- | --- | --- | --- | --- | --- | --- | --- | --- | --- | --- | --- | --- | --- | --- | --- | --- | --- | --- | --- | --- | --- | --- | --- | --- | --- | --- | --- | --- | --- | --- | --- | --- | --- | --- | --- | --- | --- | --- | --- | --- | --- | --- | --- | --- | --- | --- | --- | --- | --- | --- | --- | --- | --- | --- | --- | --- | --- | --- | --- | --- | --- | --- | --- | --- | --- | --- | --- | --- | --- | --- | --- | --- | --- | --- | --- | --- | --- | --- | --- | --- | --- | --- | --- | --- | --- | --- | --- | --- | --- | --- | --- | --- | --- | --- | --- | --- | --- | --- | --- | --- | --- | --- | --- | --- | --- | --- | --- | --- | --- | --- | --- | --- | --- | --- | --- | --- | --- | --- | --- | --- | --- | --- | --- | --- | --- | --- | --- | --- | --- | --- | --- | --- | --- | --- | --- | --- | --- | --- | --- | --- | --- | --- | --- | --- | --- | --- | --- | --- | --- | --- | --- | --- | --- | --- | --- | --- | --- | --- | --- | --- | --- | --- | --- | --- | --- | --- | --- | --- | --- | --- | --- | --- | --- | --- | --- | --- | --- | --- | --- | --- | --- | --- | --- | --- | --- | --- | --- | --- | --- | --- | --- | --- | --- | --- | --- | --- | --- | --- | --- | --- | --- | --- | --- | --- | --- | --- | --- | --- | --- | --- | --- | --- | --- | --- | --- | --- | --- | --- | --- | --- | --- | --- | --- | --- | --- | --- | --- | --- | --- | --- | --- | --- | --- | --- | --- | --- | --- | --- | --- | --- | --- | --- | --- | --- | --- | --- | --- | --- | --- | --- | --- | --- | --- | --- | --- | --- | --- | --- | --- | --- | --- | --- | --- | --- | --- | --- | --- | --- | --- | --- | --- | --- | --- | --- | --- | --- | --- | --- | --- | --- | --- | --- | --- | --- | --- | --- | --- | --- | --- | --- | --- | --- | --- | --- | --- | --- | --- | --- | --- | --- | --- | --- | --- | --- | --- | --- | --- | --- | --- | --- | --- | --- | --- | --- | --- | --- | --- | --- | --- | --- | --- | --- | --- | --- | --- | --- | --- | --- | --- | --- | --- | --- | --- | --- | --- | --- | --- | --- | --- | --- | --- | --- | --- | --- | --- | --- | --- | --- | --- | --- | --- | --- | --- | --- | --- | --- | --- | --- | --- | --- | --- | --- | --- | --- | --- | --- | --- | --- | --- | --- | --- | --- | --- | --- | --- | --- | --- | --- | --- | --- | --- | --- | --- | --- | --- | --- | --- | --- | --- | --- | --- | --- | --- | --- | --- | --- | --- | --- | --- | --- | --- | --- | --- | --- | --- | --- | --- | --- | --- | --- | --- | --- | --- | --- | --- | --- | --- | --- | --- | --- | --- | --- | --- | --- | --- | --- | --- | --- | --- | --- | --- | --- | --- | --- | --- | --- | --- | --- | --- | --- | --- | --- | --- | --- | --- | --- | --- | --- | --- | --- | --- | --- | --- | --- | --- | --- | --- | --- | --- | --- | --- | --- | --- | --- | --- | --- | --- | --- | --- | --- | --- | --- | --- | --- | --- | --- | --- | --- | --- | --- | --- | --- | --- | --- | --- | --- | --- | --- | --- | --- | --- | --- | --- | --- | --- | --- | --- | --- | --- | --- | --- | --- | --- | --- | --- | --- | --- | --- | --- | --- | --- | --- | --- | --- | --- | --- | --- | --- | --- | --- | --- | --- | --- | --- | --- | --- | --- | --- | --- | --- | --- | --- | --- | --- | --- | --- | --- | --- | --- | --- | --- | --- | --- | --- | --- | --- | --- | --- | --- | --- | --- | --- | --- | --- | --- | --- | --- | --- | --- | --- | --- | --- | --- | --- | --- | --- | --- | --- | --- | --- | --- | --- | --- | --- | --- | --- | --- | --- | --- | --- | --- | --- | --- | --- | --- | --- | --- | --- | --- | --- | --- | --- | --- | --- | --- | --- | --- | --- | --- | --- | --- | --- | --- | --- | --- | --- | --- | --- | --- | --- | --- | --- | --- | --- | --- | --- | --- | --- | --- | --- | --- | --- | --- | --- | --- | --- | --- | --- | --- | --- | --- | --- | --- | --- | --- | --- | --- | --- | --- | --- | --- | --- | --- | --- | --- | --- | --- | --- | --- | --- | --- | --- | --- | --- | --- | --- | --- | --- | --- | --- | --- | --- | --- | --- | --- | --- | --- | --- | --- | --- | --- | --- | --- | --- | --- | --- | --- | --- | --- | --- | --- | --- | --- | --- | --- | --- | --- | --- | --- | --- | --- | --- | --- | --- | --- | --- | --- | --- | --- | --- | --- | --- | --- | --- | --- | --- | --- | --- | --- | --- | --- | --- | --- | --- | --- | --- | --- | --- | --- | --- | --- | --- | --- | --- | --- | --- | --- | --- | --- | --- | --- | --- | --- | --- | --- | --- | --- | --- | --- | --- | --- | --- | --- | --- | --- | --- | --- | --- | --- | --- | --- | --- | --- | --- | --- | --- | --- | --- | --- | --- | --- | --- | --- | --- | --- | --- | --- | --- | --- | --- | --- | --- | --- | --- | --- | --- | --- | --- | --- | --- | --- | --- | --- | --- | --- | --- | --- | --- | --- | --- | --- | --- | --- | --- | --- | --- | --- | --- | --- | --- | --- | --- | --- | --- | --- | --- | --- | --- | --- | --- | --- | --- | --- | --- | --- | --- | --- | --- | --- | --- | --- | --- | --- | --- | --- | --- | --- | --- | --- | --- | --- | --- | --- | --- | --- | --- | --- | --- | --- | --- | --- | --- | --- | --- | --- | --- | --- | --- | --- | --- | --- | --- | --- | --- | --- | --- | --- | --- | --- | --- | --- | --- | --- | --- | --- | --- | --- | --- | --- | --- | --- | --- | --- | --- | --- | --- | --- | --- | --- | --- | --- | --- | --- | --- | --- | --- | --- | --- | --- | --- | --- | --- | --- | --- | --- | --- | --- | --- | --- | --- | --- | --- | --- | --- | --- | --- | --- | --- | --- | --- | --- | --- | --- | --- | --- | --- | --- | --- | --- | --- | --- | --- | --- | --- | --- | --- | --- | --- | --- | --- | --- | --- | --- | --- | --- | --- | --- | --- | --- | --- | --- | --- | --- | --- | --- | --- | --- | --- | --- | --- | --- | --- | --- | --- | --- | --- | --- | --- | --- | --- | --- | --- | --- | --- | --- | --- | --- | --- | --- | --- | --- | --- | --- | --- | --- | --- | --- | --- | --- | --- | --- | --- | --- | --- | --- | --- | --- | --- | --- | --- | --- | --- | --- | --- | --- | --- | --- | --- | --- | --- | --- | --- | --- | --- | --- | --- | --- | --- | --- | --- | --- | --- | --- | --- | --- | --- | --- | --- | --- | --- | --- | --- | --- | --- | --- | --- | --- | --- | --- | --- | --- | --- | --- | --- | --- | --- | --- | --- | --- | --- | --- | --- | --- | --- | --- | --- | --- | --- | --- | --- | --- | --- | --- | --- | --- | --- | --- | --- | --- | --- | --- | --- | --- | --- | --- | --- | --- | --- | --- | --- | --- | --- | --- | --- | --- | --- | --- | --- | --- | --- | --- | --- | --- | --- | --- | --- | --- | --- | --- | --- | --- | --- | --- | --- | --- | --- | --- | --- | --- | --- | --- | --- | --- | --- | --- | --- | --- | --- | --- | --- | --- | --- | --- | --- | --- | --- | --- | --- | --- | --- | --- | --- | --- | --- | --- | --- | --- | --- | --- | --- | --- | --- | --- | --- | --- | --- | --- | --- | --- | --- | --- | --- | --- | --- | --- | --- | --- | --- | --- | --- | --- | --- | --- | --- | --- | --- | --- | --- | --- | --- | --- | --- | --- | --- | --- | --- | --- | --- | --- | --- | --- | --- | --- | --- | --- | --- | --- | --- | --- | --- | --- | --- | --- | --- | --- | --- | --- | --- | --- | --- | --- | --- | --- | --- | --- | --- | --- | --- | --- | --- |

**Supplemental Table S5** List of 28 genes associated with CRC prognosis.

| Gene | HR | HR.95L | HR.95H | pvalue | FDR-pvalue |
| --- | --- | --- | --- | --- | --- |
| KCNE3 | 0.640493 | 0.465336 | 0.881581 | 0.006272 | 0.047448 |
| ATP2A3 | 0.744404 | 0.597636 | 0.927216 | 0.008428 | 0.047448 |
| TTLL4 | 0.531554 | 0.3319 | 0.851309 | 0.008541 | 0.047448 |
| TOM1 | 1.796764 | 1.149469 | 2.808567 | 0.010135 | 0.047448 |
| FBN2 | 0.785591 | 0.653578 | 0.944267 | 0.010143 | 0.047448 |
| ZNF333 | 0.643048 | 0.454587 | 0.90964 | 0.01259 | 0.047448 |
| NFKBIA | 1.802326 | 1.116822 | 2.908592 | 0.015846 | 0.047448 |
| TET3 | 0.630581 | 0.432663 | 0.919034 | 0.016427 | 0.047448 |
| DPYSL2 | 0.697709 | 0.514426 | 0.946292 | 0.020613 | 0.047448 |
| LTBP1 | 1.371035 | 1.049197 | 1.791594 | 0.020789 | 0.047448 |
| RASSF4 | 1.519407 | 1.065633 | 2.16641 | 0.020823 | 0.047448 |
| TNFRSF12A | 1.458564 | 1.05757 | 2.011602 | 0.021379 | 0.047448 |
| FTH1 | 1.456092 | 1.051275 | 2.016794 | 0.02377 | 0.047448 |
| NFE2 | 0.924513 | 0.863458 | 0.989885 | 0.024347 | 0.047448 |
| FRAT1 | 0.767556 | 0.608636 | 0.967972 | 0.025419 | 0.047448 |
| TESC | 0.86605 | 0.762007 | 0.984298 | 0.027642 | 0.048373 |
| ZFAND3 | 0.461682 | 0.22608 | 0.942807 | 0.033869 | 0.049774 |
| CDC42EP4 | 0.551602 | 0.317944 | 0.956976 | 0.03431 | 0.049774 |
| PLIN2 | 1.349918 | 1.022304 | 1.782522 | 0.034388 | 0.049774 |
| FUOM | 1.184503 | 1.01073 | 1.388152 | 0.036455 | 0.049774 |
| TRAPPC10 | 0.625083 | 0.397797 | 0.982232 | 0.041579 | 0.049774 |
| IMPA2 | 0.700612 | 0.496646 | 0.988345 | 0.042688 | 0.049774 |
| RELL2 | 0.88881 | 0.792555 | 0.996755 | 0.043848 | 0.049774 |
| COTL1 | 1.458331 | 1.009674 | 2.106352 | 0.044295 | 0.049774 |
| SUFU | 0.563699 | 0.320854 | 0.990346 | 0.046185 | 0.049774 |
| SPTSSA | 1.637188 | 1.00714 | 2.661385 | 0.046739 | 0.049774 |
| DESI1 | 1.762757 | 1.000882 | 3.104571 | 0.049644 | 0.049774 |
| ZFAND5 | 1.851526 | 1.000608 | 3.426066 | 0.049774 | 0.049774 |
